# Identification and Validation of an 11-Ferroptosis Related Gene Signature and Its Correlation With Immune Checkpoint Molecules in Glioma

**DOI:** 10.3389/fcell.2021.652599

**Published:** 2021-06-23

**Authors:** Zhuohui Chen, Tong Wu, Zhouyi Yan, Mengqi Zhang

**Affiliations:** Department of Neurology, Xiangya Hospital, Central South University, Changsha, China

**Keywords:** ferroptosis, tumor immunity, prognosis, gene signature, glioma

## Abstract

**Background:**

Glioma is the most common primary malignant brain tumor with significant mortality and morbidity. Ferroptosis, a novel form of programmed cell death (PCD), is critically involved in tumorigenesis, progression and metastatic processes.

**Methods:**

We revealed the relationship between ferroptosis-related genes and glioma by analyzing the mRNA expression profiles from The Cancer Genome Atlas (TCGA), Chinese Glioma Genome Atlas (CGGA), GSE16011, and the Repository of Molecular Brain Neoplasia Data (REMBRANDT) datasets. The least absolute shrinkage and selection operator (LASSO) Cox regression analysis was performed to construct a ferroptosis-associated gene signature in the TCGA cohort. Glioma patients from the CGGA, GSE16011, and REMBRANDT cohorts were used to validate the efficacy of the signature. Receiver operating characteristic (ROC) curve analysis was applied to measure the predictive performance of the risk score for overall survival (OS). Univariate and multivariate Cox regression analyses of the 11-gene signature were performed to determine whether the ability of the prognostic signature in predicting OS was independent. Gene Ontology (GO) analysis and Kyoto Encyclopedia of Genes and Genomes (KEGG) pathway analysis were conducted to identify the potential biological functions and pathways of the signature. Subsequently, we performed single sample gene set enrichment analysis (ssGSEA) to explore the correlation between risk scores and immune status. Finally, seven putative small molecule drugs were predicted by Connectivity Map.

**Results:**

The 11-gene signature was identified to divide patients into two risk groups. ROC curve analysis indicated the 11-gene signature as a potential diagnostic factor in glioma patients. Multivariate Cox regression analyses showed that the risk score was an independent predictive factor for overall survival. Functional analysis revealed that genes were enriched in iron-related molecular functions and immune-related biological processes. The results of ssGSEA indicated that the 11-gene signature was correlated with the initiation and progression of glioma. The small molecule drugs we selected showed significant potential to be used as putative drugs.

**Conclusion:**

we identified a novel ferroptosis-related gene signature for prognostic prediction in glioma patients and revealed the relationship between ferroptosis-related genes and immune checkpoint molecules.

## Introduction

Glioma is the most common primary malignant intracranial tumor. Glioblastoma, the most malignant form (WHO grade IV glioma, GBM), has a 5-year survival rate of less than 5% (Ostrom et al., 2014; Gusyatiner and Hegi, 2018). Though low-grade gliomas have a better prognosis than glioblastomas, 70% of the patients inevitably develop into glioblastomas within 10 years, posing the importance of early diagnosis and risk assessment to improve the prognosis of gliomas (Kiran et al., 2019). The rapid progression, along with the highly heterogeneous nature of gliomas, makes prognostic prediction challenging. Standard treatment of gliomas involves observation, surgery, chemotherapy, and radiotherapy (Wang and Mehta, 2019). Despite significant advances in glioma management over the past decades leading to remarkable improvements in overall survival, treatment of gliomas remains a challenge because of heterogeneity, highly proliferative rate and the infiltrative nature of the tumor cells (Delgado-López et al., 2017; Li and Ding, 2017; Esparragosa et al., 2018). All these malignant biological features make gliomas highly recurrent and drug-resistant. Recently discovered biomarkers indicate improved survival and specific antitumor treatment (Ostrom et al., 2014; Esparragosa et al., 2018). Numerous clinical trials targeting these molecule markers for glioma therapies have been carried out, but few have ultimately succeeded. Therefore, identifying novel and effective prognostic models and drug targets is an urgent and critical task not only for glioma management, but also for drug discovery.

Ferroptosis, first proposed by Dixon in 2012, is a newly discovered type of programmed cell death (PCD) that occurs through Fe(II)-dependent lipid peroxidation due to insufficient cellular reducing capacity (Dixon et al., 2012; Fearnhead et al., 2017; Stockwell et al., 2017). Previous studies have shown that ferroptosis is closely related to the progression of tumors, such as hepatocellular carcinoma (HCC), renal cell carcinoma, adrenocortical carcinomas, ovarian cancer and pancreatic carcinoma (Belavgeni et al., 2019; Xia et al., 2019). Accumulating evidence has demonstrated that ferroptosis has emerged as a promising target in cancer therapeutics, especially for malignancies resistant to traditional treatments (Hassannia et al., 2019; Liang et al., 2019). Numerous genes have been identified as mediators or modulators of ferroptosis. Ferroptotic regulatory genes such as TP53 (Cao and Dixon, 2016), BAP1 (Di Nunno et al., 2019), GPX4 (Liu et al., 2018), and DPP4 (Enz et al., 2019) are critically involved in tumorigenesis and progression. However, whether these genes correlate with the prognosis of glioma patients has yet to be elucidated.

In the current study, we first identified the differential expression of ferroptosis-related genes in glioma samples according to publicly accessible mRNA expression profiles and corresponding clinical data of glioma patients. Then, we constructed a gene-based prognostic model in the TCGA dataset and validated the signature of ferroptosis-related genes in the CGGA dataset. We further performed the functional annotation to explore the underlying mechanisms. Finally, we selected several small molecule drugs as potential therapeutic target for glioma.

## Materials and Methods

**FIGURE 1:**
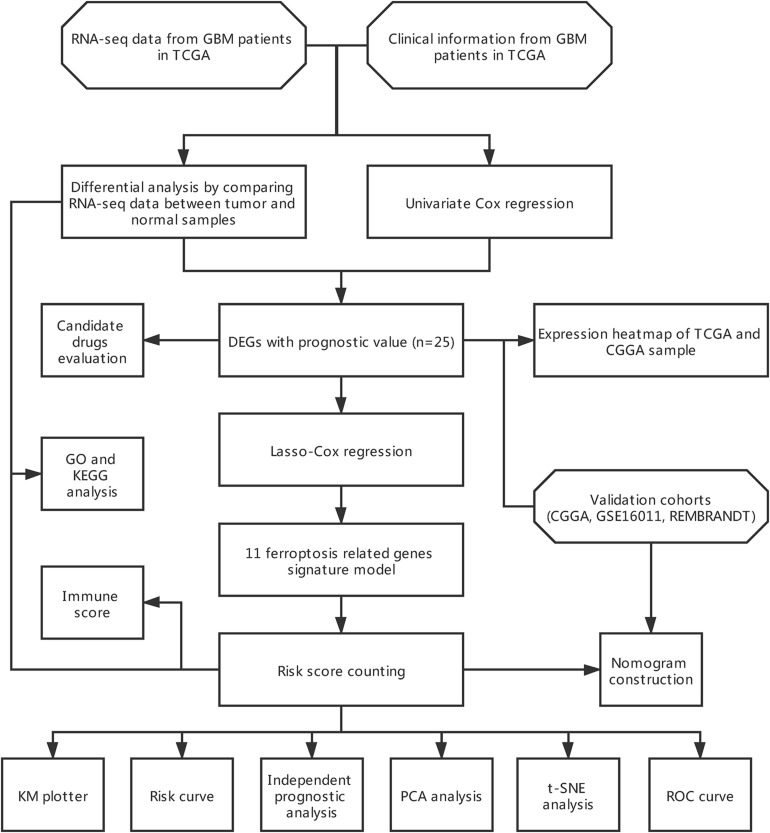
Flow chart of the study.

The flow chart of data collection and analysis is shown in Figure 1.

### Data Acquisition

In our current study, we collected four public cohorts containing RNA-seq data (expression matrix has been transformed to TPM) and clinical information of patients obtained from The Cancer Genome Atlas (TCGA)^1^, Chinese Glioma Genome Atlas (CGGA)^2^ database, GEO^3^, and GlioVis^4^. TCGA-LGG (Brat et al., 2015) and TCGA-GBM (Brennan et al., 2013) combined (*n* = 703) were used to find aberrantly expressed genes between cancer and normal tissue, based on previously published ferroptosis related genes, and to construct the prognostic signature model (Stockwell et al., 2017; Bersuker et al., 2019; Doll et al., 2019; Hassannia et al., 2019). Basically, we chose the mentioned protein-coding genes from these studies and combined them as ferroptosis related genes. The retrieved genes were listed in Supplementary Table 1. CGGA dataset (Zhao et al., 2017) (*n* = 325), GSE16011 (Gravendeel et al., 2009) (*n* = 276) and Repository of Molecular Brain Neoplasia Data (REMBRANDT) (Madhavan et al., 2009) (*n* = 444) were used as validation cohorts to assess the efficacy of our gene signature model.

### Identification of Differentially Expressed Genes (DEGs)

DEGs were identified by comparing the mRNA expression of 60 ferroptosis related genes between tumor and normal tissue in TCGA datasets using Limma package version 3.44.3 in R software version 4.0.3. The information of sample sources (TCGA-LGG or TCGA-GBM) was included as covariates during the analysis. Genes with *p* < 0.05 were selected for further analysis. The DEGs between low- and high-risk group were also identified in both TCGA and CGGA cohorts after the calculation of the risk score using Limma package version 3.44.3 in R software version 4.0.3. Genes with fdr < 0.05 and |log FC| > 1 were selected for further analysis.

### Gene Correlation Analysis

The protein-protein association analysis of DEGs was performed based on the STRING database version 11.0 (von Mering et al., 2005)^5^. Genes having no predictive interaction with other DEGs were not presented in the final figure.

### Identification and Validation of Prognostic Gene Signature

Univariate Cox regression analysis was performed to determine the genes significantly associated with overall survival (OS) in TCGA datasets. Due to the existence of missing value in overall survival and survival status, only 665 patients were included in the model construction in TCGA datasets. The overlapping genes between DEGs and clinically associated genes were identified using the Venn diagram. After that, the overlapping genes were sent to further construct a ferroptosis-associated gene signature by using the least absolute shrinkage and selection operator (LASSO) Cox regression analysis with the aid of Glmnet package version 4.0-2 (Tibshirani et al., 2012) in R software version 4.0.3. Based on the prognostic gene signature, the risk score for each patient can be calculated as followed:

(1) $$\text{riskScore}=\sum_{i=1}^{n}Coef\left(X_{i}\right)*Exp\left(X_{i}\right)$$

In formula (1), *Coef*(*X*_*i*_) represented the coefficient of each ferroptosis-related genes *X_i_*, and *Exp*(*X*_*i*_) represented the expression levels of these genes. The calculated risk score divided all the patients into low- or high-risk using median risk score as a cutoff. The risk score of patients from CGGA datasets can also be calculated to validate the efficacy of the prognostic gene signature.

### Gene Enrichment Analysis

To functionally annotate differentially expressed gene sets during the analysis, Gene Ontology (GO) and Kyoto Encyclopedia of Genes and Genomes (KEGG) pathway analysis were performed in R software version 4.0.3 using ClusterProfiler version 3.16.1 (Yu et al., 2012) package. PFAM Protein Domains, INTERPRO Protein Domains and Features enrichment were performed directly in the STITCH database version 5.0^6^ (Szklarczyk et al., 2016). To estimate the immune cell infiltration and immune function status in high-risk patients vs. low-risk ones, single sample gene set enrichment analysis (ssGSEA) was performed using GSVA version 1.36.3 (Hänzelmann et al., 2013) package in R software version 4.0.3. In short, the immune-related gene set enrichment score of each patient was first calculated. Then the patients were divided into high- or low-risk groups based on the formerly mentioned cutoff, after which the immune status was compared between the two groups.

### Nomogram Construction

The independent clinical factor validated by univariate and multivariate Cox regression analysis were enrolled to construct a nomogram for prognosis prediction, which included primary/recurrent/secondary (PRS) type of glioma, grade, 1p19q codeletion status and risk score. Patients with missing data were excluded from the analysis and thus only 275 patients were included in the univariate and multivariate Cox regression analysis. Package Rms version 6.1-0 was utilized to perform the construction and calculate the concordance index (C-index) to evaluate the model efficacy in prognosis prediction. The closer its value is to 1, the better the performance. In addition, calibration curves for 1–, 2–, and 3– year prediction were plotted to assess the consistency between predicted and actual survival.

### Candidate Small Molecule Drugs Analysis and Downstream Target Molecule Identification

The Connectivity Map (CMap) database version build 02^7^ was used to predict the putative drugs targeting DEGs in our present analysis. CMap database (Lamb et al., 2006) can be utilized to explore functional links between disease, genetic interference and drug action. The enrichment scores ranging from –1 to 1 were calculated for each putative drugs. The negative enrichment score of a drug represented the reversing effects on the input gene set, thus indicating its anti-tumor capacity when it comes to cancer-related gene set. Besides, percent non-null represented the percentage of meaningful results obtained in the whole n times experiments conducted by CMap database. The small molecule compounds were chosen with *p* < 0.05 and enrichment scores <–0.85. Subsequently, all the small molecule compounds with *p* < 0.05 were collected and analyzed in the STITCH database version 5.0 (see text footnote 6) to identify the target proteins and mechanism of action. The STITCH database (Szklarczyk et al., 2016) is a platform for searching known and predicted interactions between drug compounds and proteins. The interactions between drug compounds and proteins are verified through experiments, databases, and studies in the literature.

### Statistical Analysis

All the data were analyzed using the R software version 4.0.3^8^. Kaplan-Meier analysis was performed to compare the overall survival curves between different patient groups, with a log-rank test to evaluate the statistical significance. Spearman’s rank correlation coefficient was calculated to evaluate the linear correlation between risk score and the expression of immune checkpoint related genes. Kruskal-Wallis H-test was used to compare difference between groups. *p* < 0.05 was considered statistically significant for all the analyses.

## Results

### Identification of 25 Prognostic Ferroptosis-Related DEGs in the TCGA Cohort

**FIGURE 2:**
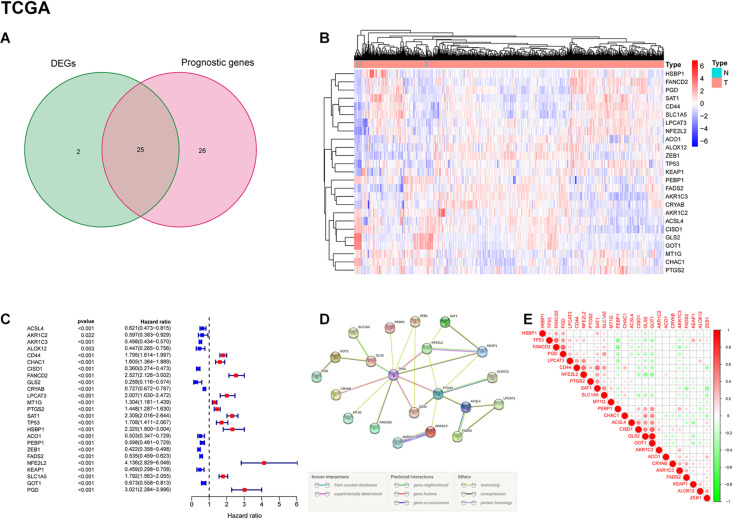
Identification of prognostic ferroptosis-related DEGs in the TCGA cohort. **(A)** Venn diagram shows the differentially expressed genes between tumor and adjacent normal tissues of prognostic value. **(B)** Gene expression levels of DEGs in the TCGA dataset. Red indicates up-regulated genes; blue indicates down-regulated genes. **(C)** Results of univariate Cox regression analysis between candidate gene expression and OS. **(D)** PPI network among candidate genes indicates that TP53 and PTGS2 are the hub genes. **(E)** The correlation between candidate genes. Red represents positive correlation; green represents negative correlation.

A total of 703 GBM patients from two TCGA cohorts (including TCGA-GBM and TCGA-LGG) and 325 GBM patients from CGGA cohort were included in the analysis. To evaluate the expression differences of ferroptosis-related genes between tumor tissues and adjacent normal tissues, we analyzed RNA-seq data from TCGA dataset. Subsequently, 27 of 60 ferroptosis-related genes were selected, and 25 of them were related to patient survival (Figure 2A). Among the 25 overlapping genes, HSBP1, FANCD2, PGD, SAT1, CD44, SLC1A5, LPCAT3, NFE2L2, ACO1, ALOX12, ZEB1, TP53, KEAP1, PEBP1, FADS2, AKR1C3, and CRYAB were upregulated, and AKR1C2, ACSL4, CISD1, GLS2, GOT1, MT1G, CHAC1, and PTGS2 were downregulated in tumor tissues (Figure 2B). The results of univariate Cox regression analysis between gene expression and OS is shown in Figure 2C (all *p* < 0.05). The protein-protein interaction (PPI) network and functional analysis of these genes indicated that TP53 and PTGS2 were the hub genes (Figure 2D). The correlation of candidate genes is presented in Figure 2E.

### Construction of a Gene-Based Prognostic Model in the TCGA Cohort

**FIGURE 3:**
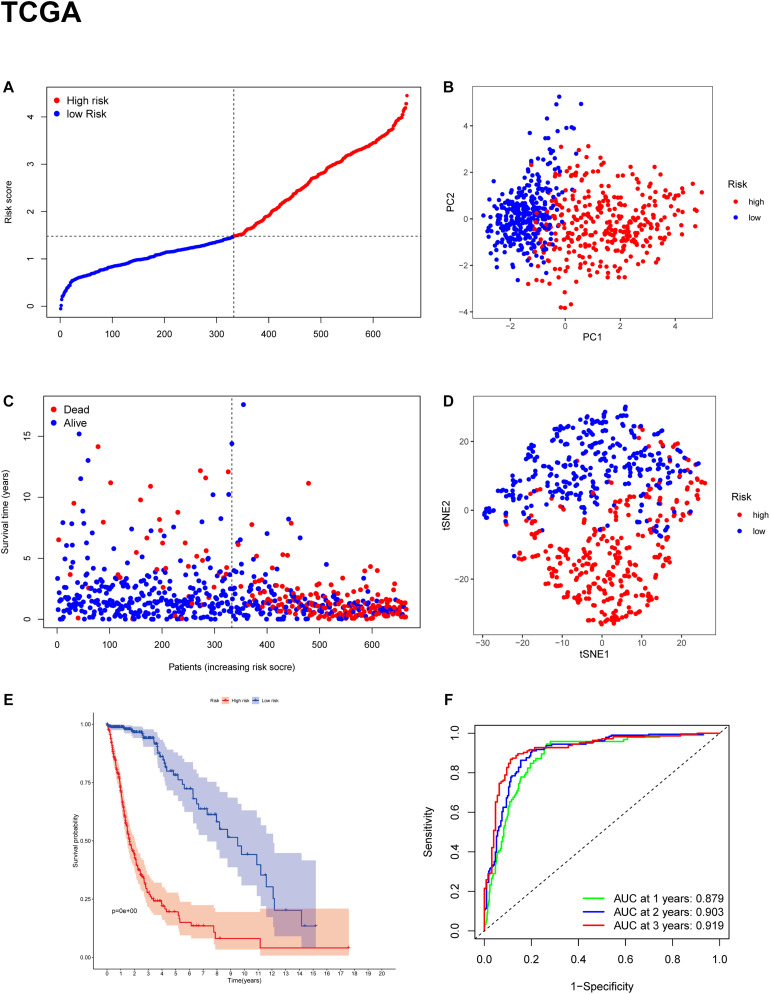
Construction of the gene signature in the TCGA cohort. **(A,C)** Distribution and median value of risk scores in the TCGA cohort. **(B)** PCA plot of the TCGA cohort. **(D)** t-SNE analysis of the TCGA cohort. **(E)** Kaplan-Meier survival curve for the OS of patients in the high-risk group (red line) and low-risk group (blue line) in the TCGA cohort. **(F)** AUC of time-dependent ROC curve analysis for evaluating the prognostic performance of the risk score for OS in the TCGA cohort.

To evaluate the risk of each patient, LASSO-Cox regression analysis was applied to establish a gene-based prognostic model using the expression profile of the 25 genes mentioned above. We identified an 11-gene signature based on the LASSO regression with the optimal value of λ and performed survival analyses according to the optimal cut-off expression value of each gene (Supplementary Figure 1). The results indicated that high expression of CD44, FANCD2, HSBP1, MT1G, NFE2L2, SAT1, and low expression of AKR1C3, ALOX12, CRYAB, FADS2, and ZEB1 correlated with a poor prognosis. The calculated coefficient was exhibited in Table 1. The distribution of risk scores in the TCGA dataset is presented in Figure 3A. The patients were divided into a high-risk group and a low-risk group based on the median cut-off value. Principal component analysis (PCA) and t-distributed stochastic neighbor embedding (t-SNE) analysis were used to separate between the two different risk groups (Figures 3B,D). The result indicated that patients in high-risk group and low-risk group were distributed in discrete directions. The OS status in the high-risk group was significantly poorer than that in the low-risk group (Figure 3C). Consistently, Kaplan-Meier survival curve in the TCGA cohort indicated that a high-risk score was correlated with a worse prognosis (Figure 3E, *p* < 0.001). Time-dependent ROC curve analysis was applied to measure the predictive performance of the risk score for OS. The signature of the 11 ferroptosis-related genes exhibited remarkable prognostic validity, with the area under the curve (AUC) reaching 0.879 (0.843–0.915, 95%CI) at 1 year, 0.903 (0.871–0.935, 95%CI) at 2 years, and 0.919 (0.884–0.954, 95%CI) at 3 years (Figure 3F).

**TABLE 1:** The calculated coefficient of the 11 ferroptosis-related genes.

Gene	Coefficient
AKR1C3	–0.120098473470357
ALOX12	–0.0122009713910145
CD44	0.214177710154476
FANCD2	0.684183489406273
CRYAB	–0.104571744619555
MT1G	0.0523002991814781
SAT1	0.17208169582249
HSBP1	0.0301696571931318
ZEB1	–0.371498258625237
FADS2	–0.0378977482917486
NFE2L2	0.452805076174773

### Validation of the Signature of 11 Ferroptosis-Related Genes in the CGGA, GSE16011, and REMBRANDT Cohorts

**FIGURE 4:**
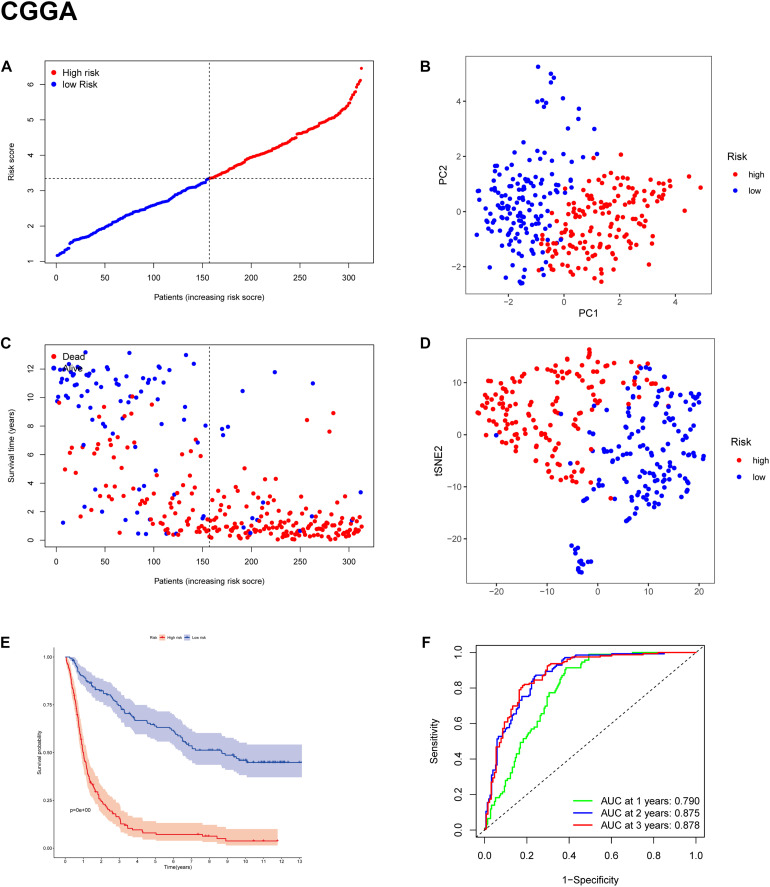
Prognostic validation of the 11-gene signature in the CGGA cohort. **(A,C)** Distribution and median value of risk scores in the CGGA cohort. **(B)** PCA plot of the CGGA cohort. **(D)** t-SNE analysis of the CGGA cohort. **(E)** Kaplan-Meier survival curve for the OS of patients in the high-risk group (red line) and low-risk group (blue line) in the CGGA cohort. **(F)** AUC of time-dependent ROC curve analysis in the CGGA cohort.

**FIGURE 5:**
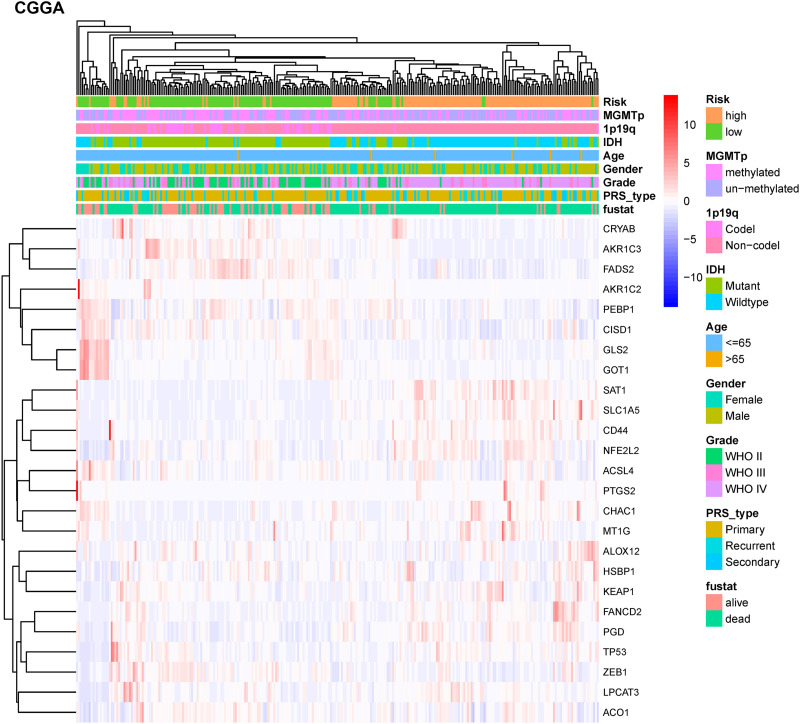
Hierarchical clustering showing correlation between signature risk score, different expression levels of selected ferroptosis-related genes, and clinical or molecular features in the CGGA dataset. Heatmap showed the different expression levels of 25 ferroptosis-related genes and clinical or molecular pathological features using hierarchical clustering in CGGA dataset. Methylguanine methyltransferase promotor (MGMTp); isocitrate dehydrogenase (IDH); co-deletion (Codel); without co-deletion (Non-codel); primary/recurrent/secondary type of glioma (PRS_type); survival status (fustat).

To test the robustness of the model constructed by the TCGA cohort, we performed prognostic analyses in the CGGA, GSE16011, and REMBRANDT validation cohorts. In the CGGA cohort, the patients were also divided into high-risk and low-risk groups according to the median value with the same calculation formula as the TCGA cohort (Figure 4A). Likewise, PCA and t-SNE analysis in the CGGA cohort confirmed that patients in high-risk group and low-risk group were distributed in two directions (Figures 4B,D). Similar to the results from the TCGA cohort, patients with a high-risk score were more likely to have a significantly shorter OS and poorer prognosis in the CGGA dataset (Figures 4C,E). The AUC of the signature was 0.790 (0.741–0.839, 95%CI) at 1 year, 0.875 (0.835–0.915, 95%CI) at 2 years, and 0.878 (0.836–0.919, 95%CI) at 3 years (Figure 4F). The GSE16011 cohort (Supplementary Figure 2) and the REMBRANDT cohort (Supplementary Figure 3) exhibited a pattern similar to the CGGA cohort. Heat maps showed clinical and molecular features and different expression levels of 25 selected genes using hierarchical clustering in the TCGA dataset (Supplementary Figure 4) and CGGA dataset (Figure 5). Consistent with the calculated risk score, patients were roughly clustered into two groups by risk score. In the CGGA dataset, with an increase in risk score, the expression levels of CRYAB, AKR1C3, FADS2, AKR1C2, PEBP1, CISD1, GLS2, and GOT1 were downregulated; the expression levels of SAT1, SLC1A5, CD44, NFE2L2, ACSL4, PTGS2, CHAC1, MT1G, ALOX12, HSBP1, KEAP1, FANCD2, PGD, TP53, ZEB1, LPCAT3, and ACO1 were upregulated. Clinical and molecular features, such as methylated MGMTp, 1p19q non-codeletion, IDH wild types, recurrent/secondary tumor types were enriched in high-risk-score samples. In the TCGA dataset, there are only 443 samples with both clinical data and RNAseq data, so blank and missing data existed when clinical parameters were added to the heatmap. The incomplete clinical data may not be completely random, leading to bias in the clinical correlation analysis. The result in the TCGA dataset was basically consistent with CGGA dataset. These results indicated that the risk score of the ferroptosis-related gene signatures positively correlated with glioma.

### Independent Prognostic Value of the 11-Gene Signature

**FIGURE 6:**
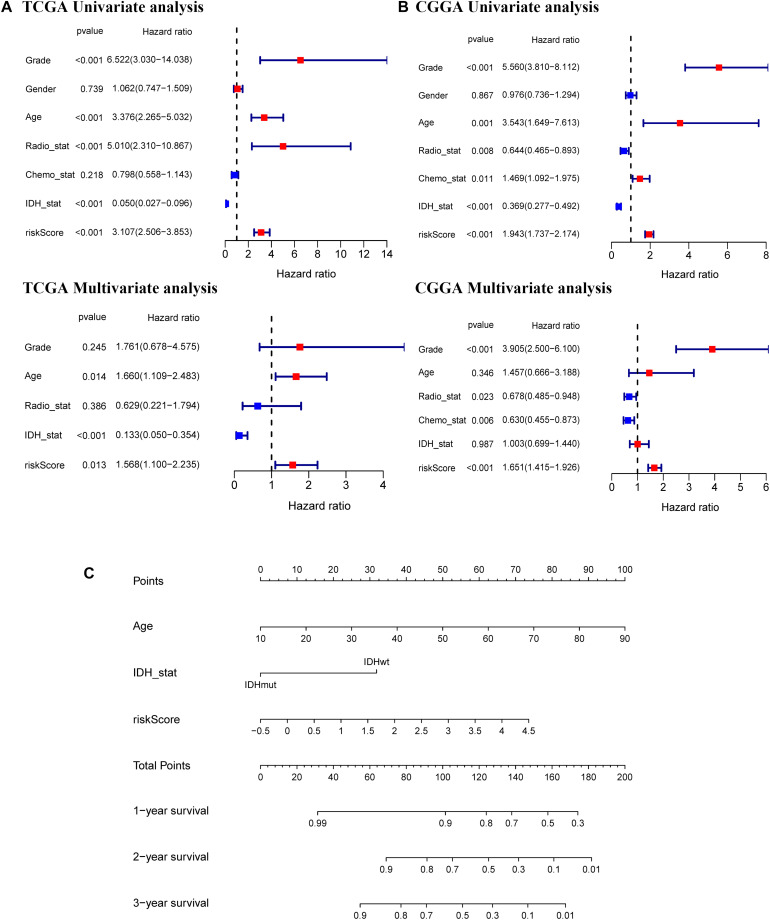
Independent prognostic value of the 11-gene signature in the TCGA and CGGA cohorts and construction of the predictive nomogram from the CGGA cohort. Univariate and multivariate Cox regression analyses of the signature in the TCGA derivation cohort **(A)** and the CGGA validation cohort **(B)**. **(C)** A nomogram of the 11-gene signature for predicting 3-year survival in the TCGA dataset.

Univariate and multivariate Cox regression analyses of the 11-gene signature were performed in the TCGA, CGGA, GSE16011, and REMBRANDT datasets to determine whether the ability of the prognostic signature in predicting OS was independent. The results of univariate Cox regression analyses determined that the risk score was significantly related to OS in both the TCGA cohort and the CGGA cohort (TCGA cohort: *HR* = 3.107, 95% CI = 2.506–3.853, *p* < 0.001; CGGA cohort: *HR* = 1.943, 95% CI = 1.737–2.174, *p* < 0.001) (Figures 6A,B). In multivariate Cox regression analyses, the risk score still proved to be an independent predictive factor for OS (TCGA cohort: *HR* = 1.568, 95% CI = 1.100–2.235, *p* < 0.05; CGGA cohort: *HR* = 1.651, 95% CI = 1.415–1.926, *p* < 0.001) (Figures 6A,B). These consistent results were also validated in the GSE16011 and REMBRANDT datasets (Supplementary Figure 5). Based on the independent prognostic parameters for the OS in the TCGA dataset, we constructed a nomogram to predict 1, 2, and 3-year survival (Figure 6C). Besides, the calibration curve for the probability of 1, 2, and 3-year OS showed an optimal agreement between observation and prediction in the TCGA dataset (Supplementary Figure 6). The C-index was calculated as 0.83 after bias correction, showing relatively high performance in clinical diagnosis. Therefore, these results indicated that the 11-gene signature may serve as a potential diagnostic factor in glioma patients. Meanwhile, the above findings also provided us with useful information that upregulated expression of ferroptosis-related genes has an impact on the prognosis of glioma.

### Functional Annotation of the 11-Gene Signature

**FIGURE 7:**
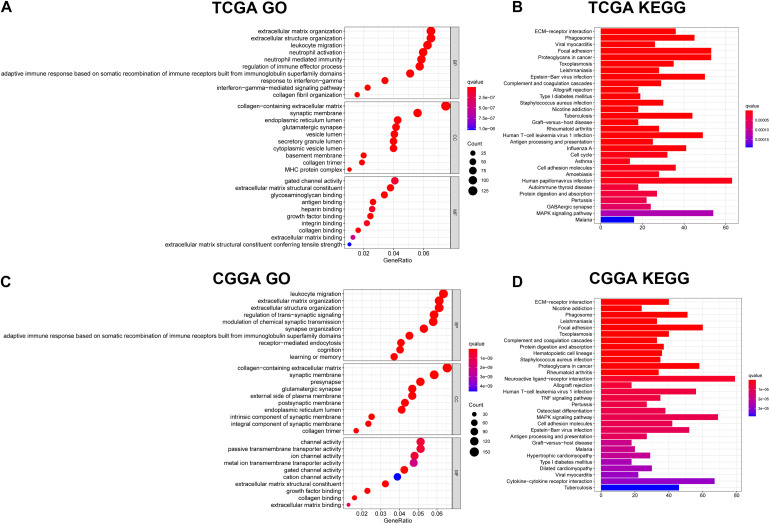
Results of GO and KEGG analyses. **(A)** GO analysis in the TCGA dataset. **(B)** KEGG pathway analysis in the TCGA dataset. **(C)** GO analysis in the CGGA dataset. **(D)** KEGG pathway analysis in the CGGA dataset.

To identify the potential biological functions and pathways of the 11-gene signature, the DEGs between the high-risk group and the low-risk group were used to conduct GO analysis and KEGG pathway analysis. Based on GO analysis, the DEGs were enriched in iron-related molecular functions, such as gated channel activity in the TCGA and CGGA cohorts, and channel activity and ion channel activity in the CGGA cohort. In addition, DEGs in the TCGA cohort were highly enriched in several immune-related biological processes, such as adaptive immune response based on somatic recombination of immune receptors built from immunoglobulin superfamily domains, leukocyte migration, interferon-gamma-mediated signaling pathway, response to interferon-gamma, regulation of immune effector process, neutrophil activation and neutrophil mediated immunity (Figure 7A). Two immune-related biological processes were validated in the CGGA cohort, including leukocyte migration and adaptive immune response based on somatic recombination of immune receptors built from immunoglobulin superfamily domains (Figure 7C). KEGG pathway analysis indicated that DEGs were enriched in immune-related pathways, which was consistent with the results of GO analysis. The terms included phagosome, complement and coagulation cascades, allograft rejection, human T-cell leukemia virus 1 infection, antigen processing and presentation, cell adhesion molecules and graft-vs.-host disease in both the TCGA dataset (Figure 7B) and the CGGA dataset (Figure 7D), TNF signaling pathway and cytokine-cytokine receptor interaction in the CGGA dataset (Figure 7D). Besides, several cancer-related terms were included in the two datasets, such as proteoglycans in cancer and Epstein-Barr virus infection in the TCGA and CGGA datasets, and human papillomavirus infection in the TCGA dataset. The aforementioned results indicate that the 11-gene signature highly correlates with cancer progression, particularly by affecting immune-related functions.

### The Ferroptosis Related Gene Signature Is Highly Correlated With Immune Function and Immune Checkpoint Molecules in Glioma

**FIGURE 8:**
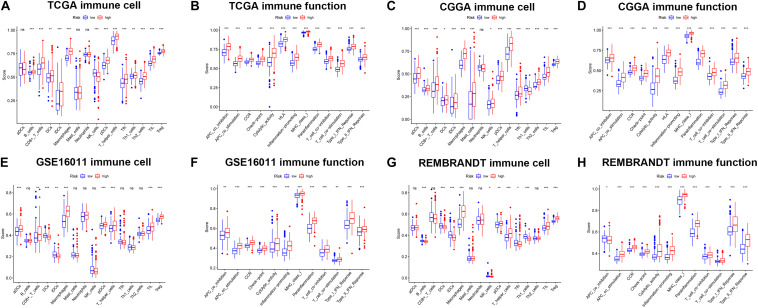
The ssGSEA results of different risk groups in the TCGA cohort **(A,B)**, CGGA cohort **(C,D)**, GSE16011 cohort **(E,F)**, and REMBRANDT cohort **(G,H)**. The scores of 16 immune cells **(A,C,E,G)** and 13 immune-related functions **(B,D,F,H)** were shown in boxplots. Adjusted p were showed as: ns, not significant; *p < 0.05; **p < 0.01; ***p < 0.001.

To further explore the correlation between risk scores and immune status, ssGSEA was performed to quantify the enrichment scores of different immune cell subpopulations and related functions or pathways in the TCGA (Figures 8A,B), CGGA (Figures 8C,D), GSE16011 (Figures 8E,F), and REMBRANDT (Figures 8G,H) cohorts. As expected, contents of immune response had higher scores in the high-risk group, including macrophages, pDCs, T helper cells, TIL, and Treg in all datasets. The score of NK cells was lower in the high-risk group in the TCGA, GSE16011, and REMBRANDT datasets, while exhibited the opposite in the CGGA dataset. Moreover, the scores of DEGs in immune-related biological processes or molecule functions were statistically different between two risk groups in all cohorts, including APC co-inhibition, APC co-stimulation, CCR, check-point, cytolytic activity, HLA, inflammation-promoting, MHC class I, parainflammation, T cell co-inhibition, T cell co-stimulation, type I IFN response, type II IFN response. Subsequently, we calculated Spearman’s rank correlation coefficient to evaluate the linear correlation between the expression of immune checkpoint related genes (CD274, CD276, CTLA4, HAVCR2, LAG3, and PDCD1) and risk scores in the TCGA, CGGA (Supplementary Figure 7), GSE16011, and REMBRANDT (Supplementary Figure 8) datasets. The results indicated that the expression of immunosuppression-related genes had positive correlation with risk scores in all cohorts. Furthermore, in order to preliminarily determine which of the 11 genes may be the most related to immune response, we performed the correlation analysis for each ferroptosis-related gene and immune checkpoint gene (| r| > 0.4) (Supplementary Figure 9). The results revealed that key genes in the TCGA dataset were CD44, FANCD2 and SAT1, and key genes in the CGGA dataset were AKR1C3, ALOX12, CD44, SAT1, and NFE2L2. The common critical genes in the two datasets were CD44 and SAT1. A previous study has revealed that CD44 was identified as a key positive regulator of PD-L1 expression in triple-negative breast cancer and non-small cell lung cancer (Kong et al., 2020). SAT1 has been reported to contribute to p53-mediated reactive oxygen species (ROS) response and ferroptosis (Ou et al., 2016). Therefore, it can be speculated that CD44 and SAT1 may regulate immune checkpoints to involve in tumor immunity. Collectively, the results above indicated that the 11-gene signature was correlated with the initiation and progression of glioma.

### Drug Screening Using the 11-Gene Signature With CMap and STITCH Database

In order to test whether the selected 11 ferroptosis-related genes are good candidates for therapy target, CMap (the connectivity map) analysis was performed to screen small molecule drugs using the selected prognostic genes as up signature or down signature. Of the 27 prognostic genes, 18 genes with positive cox coefficient were set as the up-regulated signature and the other nine genes were set as the down-regulated signature. Seven candidate small molecule drugs (butacaine, CAY-10397, BAS-012416453, PHA-00816795, STOCK1N-35696, piperlongumine, sanguinarine) with potential value were identified with *p* < 0.05 and enrichment score <–0.85 (Table 2). Among the seven small molecule drugs, butacaine and CAY-10397 (*p* < 0.001) showed significantly negative correlation with selected mRNAs and potential to be used as putative drugs. To understand the correlation between candidate drugs and immune checkpoints, the network of interacting proteins and candidate drugs with *p* < 0.05 was constructed in the STITCH database (see text footnote 6) (Supplementary Figure 10). The results showed that the direct downstream target genes of candidate drugs included HDAC1, ESR2, CYP19A1, ESR1, AR, ADRA1A, PGR, ESRRA, NR3C1, and KCNJ11. We further performed GO, PFAM Protein Domains, INTERPRO Protein Domains and Features enrichment analyses on the above genes, and found that steroid hormone related functions were enriched, especially estrogen receptor (ER) related functions (Supplementary Table 2). A previous study has revealed that in cells with low ER expression, ferroptosis was easier to be triggered by sulfasalazine in breast cancer cells (Ou et al., 2016). Besides, the correlation between ER and tumor immunity has been also extensively researched (Kurozumi et al., 2019; Romero et al., 2020; Smida et al., 2020). Therefore, the candidate drugs targeting estrogen receptor are likely to involve in the downstream tumor immunity through ferroptosis.

**TABLE 2:** Drugs selected by CMap.

CMap name	*n*	Enrichment	*p*	Percent non-null
Butacaine	4	–0.881	0.00048	100
CAY-10397	3	–0.92	0.00092	100
BAS-012416453	3	–0.886	0.00288	100
PHA-00816795	2	–0.956	0.00421	100
STOCK1N-35696	2	–0.884	0.02696	100
Piperlongumine	2	–0.876	0.03078	100
Sanguinarine	2	–0.871	0.03306	100

## Discussion

Glioma is the most common primary malignant brain tumor characterized by rapid progression and treatment resistance, causing significant mortality and morbidity (Gusyatiner and Hegi, 2018; Liu et al., 2020). Ferroptosis is a novel form of PCD. Previous studies have identified the critical role of ferroptosis in tumorigenesis and therapies (Liu et al., 2020). In the current study, we used comprehensive bioinformatics analysis to investigate variations in expression profiling of 60 ferroptosis-related genes in glioblastoma and their association with OS. We identified the signature of 11 ferroptosis-related genes associated with progression and prognosis of GBM patients and validated the novel prognostic model in an external cohort. Functional analyses indicated that immune-related biological processes were highly enriched. In addition, we selected several small molecule compounds as potential therapeutic drugs.

The 11 prognostic genes consist of AKR1C3, ALOX12, CD44, CRYAB, FADS2, FANCD2, HSBP1, MT1G, NFE2L2, SAT1, and ZEB1. Previous studies have illustrated that most of these ferroptotic genes are critically involved in tumorigenesis, including glioma. AKR1C3 involves in steroids, prostaglandins and lipid aldehydes metabolism and plays a role in tumorigenesis of breast carcinoma, endometrial carcinoma and prostate carcinoma (Park et al., 2010). ALOX12, a hotpot of monoallelic deletion in cancers, plays an important role in p53-mediated ferroptosis. ALOX12 missense mutations diminishes polyunsaturated fatty acids oxidation and p53-mediated ferroptosis (Chu et al., 2019). CD44 is a single-pass type I transmembrane protein that has shown to be closely related to tumor development. Ferroptotic responses correlate with the transcriptional regulation of SLC7A11, a key component of the cystine-glutamate antiporter. The level of CD44 can regulate the sensitivity of tumor cells to ferroptosis and the stability of SLC7A11, a key component of the cystine-glutamate antiporter related to ferroptosis regulation (Liu et al., 2019). CRYAB, secreted via exosomes, has been reported to be up-regulated in GBM and exert anti-apoptotic activity (Kore and Abraham, 2014). FADS2 has been shown to overexpress in colorectal cancer and facilitate cancer cell proliferation by increasing the metabolism of PGE2, an oncogenic molecule associated with colorectal cancer tumorigenesis (Tian et al., 2020). Previous studies have demonstrated that FANCD2 is overexpressed in high-grade gliomas and depletion of FANCD2 may serve as a potential strategy for the treatment high-grade gliomas (Metselaar et al., 2019). HSBP1 expression has reported to be elevated in oral squamous carcinoma (OSCC) and increased HSBP1 expression enhances the sensitivity of OSCC cells in radiation (Shen et al., 2014). MT1G, a member of metallothioneins (MTs), is frequently downregulated in HCC, which can be regarded as an early event in HCC progression (Ji et al., 2014). NFE2L2 is a redox-sensitive transcriptional factor mainly located in cytoplasm. The expression of NFE2L2 has been shown to positively correlate with the expression of immune checkpoint markers in brain lower grade glioma (Ju et al., 2020). A previous study has demonstrated that SAT1 causes resistance to radiation in GBM through an shRNA screen. SAT1 also involves in cell migration, proliferation and tumor growth (Thakur et al., 2019). ZEB1 has been reported to increase in gliomas and positively correlate with tumor progression (Chen et al., 2017).

We further demonstrated that a high-risk score was associated with worse prognosis. Time-dependent ROC curve of the signature of 11 ferroptosis-related genes predicted patient OS. To investigate the correlation between the risk signature and glioma grade, we further studied the levels of risk score stratified by the grade in the TCGA, CGGA, GSE16011, and REMBRANDT cohorts (Supplementary Figure 11). The risk score increased as the grade of glioma increased. In the TCGA and CGGA datasets, WHO grade IV patients had the highest increase in the risk score, while WHO grade II patients had the lowest increase in the risk score. Patients with WHO grade III were assigned a medium risk score in both the TCGA and CGGA datasets (*p* < 0.001). In the GSE16011 and REMBRANDT datasets, due to the limited samples of patients with WHO grade I and II, the results of WHO grade I and II exhibited no statistical difference. Therefore, we reassigned the samples to WHO grade I-III and WHO grade IV, and the results were statistically different. Besides, we also plotted Kaplan-Meier curves for glioma patients with low and high risk scores classified as WHO grade II to IV in the TCGA, CGGA, GSE16011, and REMBRANDT datasets (Supplementary Figure 12). In consideration of the limited samples of patients with Grade I and II in the GSE16011 and REMBRANDT datasets, we also plotted Kaplan-Meier curves of Grade I-III. The results showed that patients with high risk had significantly shorter OS than patients with low risk in WHO grade II, WHO grade III, and WHO grade IV groups. Exceptionally, the survival plot between low- and high- risk in WHO IV patients from TCGA and REMBRANT cohorts showed no significance because there were almost no patients with WHO IV were low-risk. Similarly, the survival plot between low- and high- risk in WHO II patients from CGGA and GSE16011 cohorts showed no significance, also because there were almost no patients with WHO II were high-risk. In addition, the prognostic values based on the 11-gene signature were independent of other clinical variables, including grade, age, radiotherapy status, IDH mutation status. It can be noticed that differences existed in the prognostic value for grade, age, and IDH status in the four datasets. It is probably due to the data of the four datasets coming from people in different regions. To adjust the differences caused by populations, it requires a wider range of multi-center clinical verification. Besides, the clinical data in the TCGA cohort are incomplete, some of which have missing value. The lack of data may not be completely random, leading to bias in the clinical correlation analysis. This is also the limitation of using public data to conduct analysis. Among the 11 genes, functional analysis showed that these genes were involved in immune-related biological processes and pathways. It can be reasonably assumed that ferroptosis may be critically involved in tumor immunity. To further explore potential mechanism of the signature of 11 ferroptosis-related genes, ssGSEA analysis was performed. The enrichment scores of macrophages, pDCs, T helper cells, TIL and Treg were statistically different between two risk groups and exhibited a similar pattern in the TCGA, CGGA, GSE16011, and REMBRANDT datasets. The scores of macrophages were the most statistically different between the low risk group and the high risk group. Previous studies have reported that tumor-associated macrophages are closely related to tumor-promoting inflammation and contribute to tumor progression (Mantovani et al., 2017; Ngambenjawong et al., 2017). Plasmacytoid dendritic cells (pDCs) are an immune subgroup specialized in the production of Type I Interferons (IFNs) that critically involve in the anti-viral and anti-tumor immunity (Greene et al., 2020). However, chronic infections and cancer cause pDC functional exhaustion and inhibit pDC-derived IFN-I. In addition to the protective functions of T helper populations, they are also involved in the pathogenesis of chronic inflammatory disorders (Cosmi et al., 2014). Tumor infiltrating lymphocytes (TIL) were previously reported to play a predictive role in mediating response to chemotherapy and increasing overall survival, while the increase in immunosuppressive regulatory T-cells (Tregs) has correlation with poor prognosis (Santoiemma and Powell, 2015; Stanton and Disis, 2016). Given that high-risk group exhibited poorer prognosis, we speculated that patients with high risk may suffer pDC functional exhaustion and weakened anti-tumor immunity. Besides, the inflammatory effect of T helper cells and immunosuppression of Tregs play a dominant role in the tumor immunity, rather than the protective functions of T helper populations or TIL. Moreover, the high-risk groups in the TCGA, GSE16011, and REMBRANDT cohorts had lower fractions of NK cells, indicating that impaired antitumor immunity in patients of high-risk group may contribute to their poor prognosis. The score of NK cells between two groups in the CGGA cohort was the opposite. We speculated that the result may be due to limited samples and individual differences of the patients in the CGGA cohort. Interestingly, although high-risk groups have higher scores of functions related to antitumor immunity in both cohorts, including the activity of the type I IFN response and type II IFN response, the prognosis of the high-risk group still proves poorer than that of the low-risk group. We also noticed that cells or functions associated with immunosuppression such as the fractions of Treg cells and the activity of T cell co-inhibition in both cohorts are higher in the high-risk group. One possible speculation is that the effect of immunosuppression may play a dominant role in the immune response. Therefore, we analyzed the correlation between the expression of genes related to immunosuppression and the risk scores in the TCGA, CGGA, GSE16011, and REMBRANDT cohorts. The results indicated that the expression of immunosuppression-related genes (CD274, CD276, CTLA4, HAVCR2, LAG3, and PDCD1) had a positive correlation with risk scores in all cohorts. Notably, the expression of B7-H3 exhibited a significant correlation with risk scores, with the regression coefficient reaching 0.73 in the TCGA cohort, 0.81 in the CGGA cohort, 0.57 in the GSE16011 cohort and 0.59 in the REMBRANDT cohort. B7-H3 (CD276) is a critical immune checkpoint molecule belonging to B7-CD28 families (Picarda et al., 2016). Induced on antigen-presenting cells, B7-H3 was previously shown to act as a T cell co-inhibitor associated with diminished NFAT, NF-κB and AP-1 transcriptional factor activity (Zhang et al., 2009). Numerous studies have demonstrated that B7-H3 is highly overexpressed in human malignancies and correlates with negative prognosis and poor clinical outcome, including glioma (Wang et al., 2018; Zheng et al., 2019), HCC (Zheng et al., 2019), pancreatic cancer (Inamura et al., 2018), ovarian carcinoma (Zang et al., 2010), colorectal cancer (Ingebrigtsen et al., 2014), and bone cancer (He and Li, 2019). Therefore, B7-H3 may serve as an attractive target for immunotherapy against cancers. The immune checkpoint molecules, CTLA4 and PDCD1 (PD-1), send a negative signal to T cells, thereby suppressing effector T-cell responses (Dyck and Mills, 2017). Immunotherapy targeting immune checkpoints CTLA4 and PD-1/PD-L1 (CD274) have been used in cancer patients (Chen and Mellman, 2017; Lee et al., 2017). However, only a small portion of patients exhibit durable responses and a large number of cancers such as colorectal cancer remain largely refractory to the therapy (Chen and Mellman, 2017; Das et al., 2017). HAVCR2 (TIM-3), highly expressed on innate cells, acts as a negative regulator of Th1 and CTL responses and critically involves in tumor growth (Das et al., 2017; Joller and Kuchroo, 2017). LAG3 is also a potential target for cancer immunotherapy due to its negative regulatory role on T cells (Andrews et al., 2017). Therefore, we might suppose that the signature of 11 ferroptosis-related genes was critically involved in tumorigenesis and progression of glioma probably by regulating immune-related biological processes and pathways, which correlated with the poor prognosis of glioma. In addition, we performed a CMap analysis to screen small molecule drugs using the selected prognostic genes. Some of the candidate drugs in our results have been proven to have anti-cancer effects, including butacaine (Mizuno and Ishida, 1982), piperlongumine (Duan et al., 2016; Liu et al., 2017; Chen et al., 2019), and sanguinarine (Ma et al., 2017; Zhang et al., 2017, 2018). Piperlongumine has been reported to rapidly induce the death of human pancreatic cancer cells through, at least in part, the induction of ferroptosis (Yamaguchi et al., 2018). A previous study has revealed that sanguinarine can block PPM1A phosphatase activity to restore c-Jun N-terminal kinase (JNK) activation, resulting in increased apoptosis of M. tuberculosis (Mtb)-infected macrophages (Schaaf et al., 2017). To explore the potential mechanisms of these putative drugs, we further constructed the network of protein and drug compound interactions from the STITCH database, and found that the candidate drugs were likely to involve in tumor immunity through ferroptosis by targeting estrogen receptors. Considering that all these drugs may share similar mechanisms because of the principles of CMap database, drugs with no previous publications may also have the anti-tumor effects via targeting estrogen receptors and ferroptosis. Therefore, the 11-gene signature in the current study has the potential to be used as drug targets for therapy.

This study exists some limitations. First, all the data used to construct and validate the prognostic model in the current study were obtained from publicly available datasets. This is a retrospective study. A prospective study is needed to assess the potential application of the signature we have built to predict survival. Second, though functional analysis has revealed the correlation between the ferroptosis related gene signature and immune-related biological processes, *in vivo* and *in vitro* experiments are needed to further elucidate the specific mechanism.

## Conclusion

Our present study identified a ferroptosis related 11-genes prognostic model for glioma patients. This model proved to have relatively high efficacy and to be clinically independent of other factors, providing insight into the prediction of glioma patients’ prognosis. We also noticed that the differentially expressed ferroptosis genes were highly correlated with both pro-tumor and anti-tumor pathways, yet the latter seemed to be suppressed during the progression of glioma, indicating the putative effects of immunotherapy on glioma. Several potential drugs were also predicted based on the differentially expressed ferroptosis genes. The curative effect of the drugs and the underlying mechanisms between ferroptosis and tumor immunity in glioma remained lack of research and warranted further investigation.

## Data Availability Statement

Publicly available datasets were analyzed in this study. This data can be found here: TCGA repository, https://portal.gdc.cancer.gov/; CGGA repository, http://www.cgga.org.cn/; GEO repository, https://www.ncbi.nlm.nih.gov/geo/; and GlioVis, http://gliovis.bioinfo.cnio.es/.

## Author Contributions

ZC: conceptualization and software and formal analysis. TW: writing-original draft preparation and visualization. ZY: data curation and writing-review and editing. MZ: conceptualization, supervision, and funding acquisition and validation. All authors have read and agreed to the published version of the manuscript.

## Conflict of Interest

The authors declare that the research was conducted in the absence of any commercial or financial relationships that could be construed as a potential conflict of interest.

## Footnotes

1 https://portal.gdc.cancer.gov/

2 http://www.cgga.org.cn/

3 https://www.ncbi.nlm.nih.gov/geo/

4 http://gliovis.bioinfo.cnio.es/

5 http://string.embl.de/

6 http://stitch.embl.de/

7 https://portals.broadinstitute.org/cmap/

8 https://www.R-project.org/

## Abbreviations

TCGA: The Cancer Genome Atlas
CGGA: Chinese Glioma Genome Atlas
LASSO: least absolute shrinkage and selection operator
ROC: receiver operating characteristic
OS: overall survival
GO: Gene Ontology
KEGG: Kyoto Encyclopedia of Genes and Genomes
ssGSEA: single sample gene set enrichment analysis
GBM: glioblastoma
PCD: programmed cell death
DEGs: differentially expressed genes
PRS: primary/recurrent/secondary
C-index: concordance index
CMap: Connectivity Map
PPI: protein-protein interaction
PCA: Principal component analysis
t-SNE: t-distributed stochastic neighbor embedding
AUC: area under the curve
HR: Hazard ratio
CI: Confidence interval
aDC: Activated dendritic cell
DC: dendritic cell
iDC: Immature dendritic cell
NK: natural killer
pDC: Plasmacytoid dendritic cell
Tfh: T follicular helper cell
Th: T helper
TIL: Tumor Infiltrating Lymphocyte
Treg: T regulation
APC: Antigen presenting cell
CCR: Cytokine-cytokine receptor
HLA: Human leukocyte antigen
MHC: major histocompatibility complex
IFN: interferon
OSCC: oral squamous carcinoma
MT: metallothionein
HCC: hepatocellular carcinoma.

## References

[B1] Andrews L. P. Marciscano A. E. Drake C. G. Vignali D. A. (2017). LAG3 (CD223) as a cancer immunotherapy target. *Immunol. Rev.* 276 80–96. 10.1111/imr.12519 28258692 PMC5338468

[B2] Belavgeni A. Bornstein S. R. von Mässenhausen A. Tonnus W. Stumpf J. Meyer C. (2019). Exquisite sensitivity of adrenocortical carcinomas to induction of ferroptosis. *Proc. Natl. Acad. Sci. U.S.A.* 116 22269–22274. 10.1073/pnas.1912700116 31611400 PMC6825277

[B3] Bersuker K. Hendricks J. M. Li Z. Magtanong L. Ford B. Tang P. H. (2019). The CoQ oxidoreductase FSP1 acts parallel to GPX4 to inhibit ferroptosis. *Nature* 575 688–692. 10.1038/s41586-019-1705-2 31634900 PMC6883167

[B4] Brat D. J. Verhaak R. G. Aldape K. D. Yung W. K. Salama S. R. Cooper L. A. (2015). Comprehensive, integrative genomic analysis of diffuse lower-grade gliomas. *N. Engl. J. Med.* 372 2481–2498. 10.1056/NEJMoa1402121 26061751 PMC4530011

[B5] Brennan C. W. Verhaak R. G. McKenna A. Campos B. Noushmehr H. Salama S. R. (2013). The somatic genomic landscape of glioblastoma. *Cell* 155 462–477. 10.1016/j.cell.2013.09.034 24120142 PMC3910500

[B6] Cao J. Y. Dixon S. J. (2016). Mechanisms of ferroptosis. *Cell. Mol. Life Sci.* 73 2195–2209. 10.1007/s00018-016-2194-1 27048822 PMC4887533

[B7] Chen B. Lei Y. Wang H. Dang Y. Fang P. Wang J. (2017). Repression of the expression of TET2 by ZEB1 contributes to invasion and growth in glioma cells. *Mol. Med. Rep.* 15 2625–2632. 10.3892/mmr.2017.6288 28260066

[B8] Chen D. S. Mellman I. (2017). Elements of cancer immunity and the cancer-immune set point. *Nature* 541 321–330. 10.1038/nature21349 28102259

[B9] Chen W. Lian W. Yuan Y. Li M. (2019). The synergistic effects of oxaliplatin and piperlongumine on colorectal cancer are mediated by oxidative stress. *Cell Death Dis.* 10:600. 10.1038/s41419-019-1824-6 31395855 PMC6687721

[B10] Chu B. Kon N. Chen D. Li T. Liu T. Jiang L. (2019). ALOX12 is required for p53-mediated tumour suppression through a distinct ferroptosis pathway. *Nat. Cell Biol.* 21 579–591. 10.1038/s41556-019-0305-6 30962574 PMC6624840

[B11] Cosmi L. Maggi L. Santarlasci V. Liotta F. Annunziato F. (2014). T helper cells plasticity in inflammation. *Cytometry A* 85 36–42. 10.1002/cyto.a.22348 24009159

[B12] Das M. Zhu C. Kuchroo V. K. (2017). Tim-3 and its role in regulating anti-tumor immunity. *Immunol. Rev.* 276 97–111. 10.1111/imr.12520 28258697 PMC5512889

[B13] Delgado-López P. D. Corrales-García E. M. Martino J. Lastra-Aras E. Dueñas-Polo M. T. (2017). Diffuse low-grade glioma: a review on the new molecular classification, natural history and current management strategies. *Clin. Transl. Oncol.* 19 931–944. 10.1007/s12094-017-1631-4 28255650

[B14] Di Nunno V. Frega G. Santoni M. Gatto L. Fiorentino M. Montironi R. (2019). BAP1 in solid tumors. *Future Oncol.* 15 2151–2162. 10.2217/fon-2018-0915 31159579

[B15] Dixon S. J. Lemberg K. M. Lamprecht M. R. Skouta R. Zaitsev E. M. Gleason C. E. (2012). Ferroptosis: an iron-dependent form of nonapoptotic cell death. *Cell* 149 1060–1072. 10.1016/j.cell.2012.03.042 22632970 PMC3367386

[B16] Doll S. Freitas F. P. Shah R. Aldrovandi M. da Silva M. C. Ingold I. (2019). FSP1 is a glutathione-independent ferroptosis suppressor. *Nature* 575 693–698. 10.1038/s41586-019-1707-0 31634899

[B17] Duan C. Zhang B. Deng C. Cao Y. Zhou F. Wu L. (2016). Piperlongumine induces gastric cancer cell apoptosis and G2/M cell cycle arrest both in vitro and in vivo. *Tumour Biol.* 37 10793–10804. 10.1007/s13277-016-4792-9 26874726

[B18] Dyck L. Mills K. H. G. (2017). Immune checkpoints and their inhibition in cancer and infectious diseases. *Eur. J. Immunol.* 47 765–779. 10.1002/eji.201646875 28393361

[B19] Enz N. Vliegen G. De Meester I. Jungraithmayr W. (2019). CD26/DPP4 - a potential biomarker and target for cancer therapy. *Pharmacol. Ther.* 198 135–159. 10.1016/j.pharmthera.2019.02.015 30822465

[B20] Esparragosa I. Díez-Valle R. Tejada S. Gállego Pérez-Larraya J. (2018). Management of diffuse glioma. *Presse Med.* 47(11-12 Pt 2) e199–e212. 10.1016/j.lpm.2018.04.014 30385181

[B21] Fearnhead H. O. Vandenabeele P. Vanden Berghe T. (2017). How do we fit ferroptosis in the family of regulated cell death? *Cell Death Differ.* 24 1991–1998. 10.1038/cdd.2017.149 28984871 PMC5686356

[B22] Gravendeel L. A. M. Kouwenhoven M. C. M. Gevaert O. de Rooi J. J. Stubbs A. P. Duijm J. E. (2009). Intrinsic gene expression profiles of gliomas are a better predictor of survival than histology. *Cancer Res.* 69 9065–9072. 10.1158/0008-5472.CAN-09-2307 19920198

[B23] Greene T. T. Jo Y. R. Zuniga E. I. (2020). Infection and cancer suppress pDC derived IFN-I. *Curr. Opin. Immunol.* 66 114–122. 10.1016/j.coi.2020.08.001 32947131 PMC8526282

[B24] Gusyatiner O. Hegi M. E. (2018). Glioma epigenetics: from subclassification to novel treatment options. *Semin. Cancer Biol.* 51 50–58. 10.1016/j.semcancer.2017.11.010 29170066

[B25] Hänzelmann S. Castelo R. Guinney J. (2013). GSVA: gene set variation analysis for microarray and RNA-seq data. *BMC Bioinformatics* 14:7. 10.1186/1471-2105-14-7 23323831 PMC3618321

[B26] Hassannia B. Vandenabeele P. Vanden Berghe T. (2019). Targeting ferroptosis to iron out cancer. *Cancer Cell* 35 830–849. 10.1016/j.ccell.2019.04.002 31105042

[B27] He L. Li Z. (2019). B7-H3 and its role in bone cancers. *Pathol. Res. Pract.* 215:152420. 10.1016/j.prp.2019.04.012 31060912

[B28] Inamura K. Takazawa Y. Inoue Y. Yokouchi Y. Kobayashi M. Saiura A. (2018). Tumor B7-H3 (CD276) expression and survival in pancreatic cancer. *J. Clin. Med.* 7:172. 10.3390/jcm7070172 29996538 PMC6069252

[B29] Ingebrigtsen V. A. Boye K. Nesland J. M. Nesbakken A. Flatmark K. Fodstad Ø (2014). B7-H3 expression in colorectal cancer: associations with clinicopathological parameters and patient outcome. *BMC Cancer* 14:602. 10.1186/1471-2407-14-602 25139714 PMC4148536

[B30] Ji X. F. Fan Y. C. Gao S. Yang Y. Zhang J. J. Wang K. (2014). MT1M and MT1G promoter methylation as biomarkers for hepatocellular carcinoma. *World J. Gastroenterol.* 20 4723–4729. 10.3748/wjg.v20.i16.4723 24782625 PMC4000509

[B31] Joller N. Kuchroo V. K. (2017). Tim-3, Lag-3, and TIGIT. *Curr. Top. Microbiol. Immunol.* 410 127–156. 10.1007/82_2017_62 28900677 PMC5902028

[B32] Ju Q. Li X. Zhang H. Yan S. Li Y. Zhao Y. (2020). NFE2L2 is a potential prognostic biomarker and is correlated with immune infiltration in brain lower grade glioma: a pan-cancer analysis. *Oxid. Med. Cell. Longev.* 2020:3580719. 10.1155/2020/3580719 33101586 PMC7569466

[B33] Kiran M. Chatrath A. Tang X. Keenan D. M. Dutta A. (2019). A prognostic signature for lower grade gliomas based on expression of long non-coding RNAs. *Mol. Neurobiol.* 56 4786–4798. 10.1007/s12035-018-1416-y 30392137 PMC6499716

[B34] Kong T. Ahn R. Yang K. Zhu X. Fu Z. Morin G. (2020). CD44 promotes PD-L1 expression and its tumor-intrinsic function in breast and lung cancers. *Cancer Res.* 80 444–457. 10.1158/0008-5472.Can-19-1108 31722999

[B35] Kore R. A. Abraham E. C. (2014). Inflammatory cytokines, interleukin-1 beta and tumor necrosis factor-alpha, upregulated in glioblastoma multiforme, raise the levels of CRYAB in exosomes secreted by U373 glioma cells. *Biochem. Biophys. Res. Commun.* 453 326–331. 10.1016/j.bbrc.2014.09.068 25261722 PMC4278587

[B36] Kurozumi S. Kaira K. Matsumoto H. Hirakata T. Yokobori T. Inoue K. (2019). β(2)-Adrenergic receptor expression is associated with biomarkers of tumor immunity and predicts poor prognosis in estrogen receptor-negative breast cancer. *Breast Cancer Res. Treat.* 177 603–610. 10.1007/s10549-019-05341-6 31290053

[B37] Lamb J. Crawford E. D. Peck D. Modell J. W. Blat I. C. Wrobel M. J. (2006). The connectivity map: using gene-expression signatures to connect small molecules, genes, and disease. *Science* 313 1929–1935. 10.1126/science.1132939 17008526

[B38] Lee Y. H. Martin-Orozco N. Zheng P. Li J. Zhang P. Tan H. (2017). Inhibition of the B7-H3 immune checkpoint limits tumor growth by enhancing cytotoxic lymphocyte function. *Cell Res.* 27 1034–1045. 10.1038/cr.2017.90 28685773 PMC5539354

[B39] Li S. Ding X. (2017). TRPC channels and glioma. *Adv. Exp. Med. Biol.* 976 157–165. 10.1007/978-94-024-1088-4_14 28508321

[B40] Liang C. Zhang X. Yang M. Dong X. (2019). Recent progress in ferroptosis inducers for cancer therapy. *Adv. Mater.* 31:e1904197. 10.1002/adma.201904197 31595562

[B41] Liu D. Qiu X. Y. Wu X. Hu D. X. Li C. Y. Yu S. B. (2017). Piperlongumine suppresses bladder cancer invasion via inhibiting epithelial mesenchymal transition and F-actin reorganization. *Biochem. Biophys. Res. Commun.* 494 165–172. 10.1016/j.bbrc.2017.10.061 29037814

[B42] Liu H. Schreiber S. L. Stockwell B. R. (2018). Targeting dependency on the GPX4 lipid peroxide repair pathway for cancer therapy. *Biochemistry* 57 2059–2060. 10.1021/acs.biochem.8b00307 29584411 PMC5962875

[B43] Liu H. J. Hu H. M. Li G. Z. Zhang Y. Wu F. Liu X. (2020). Ferroptosis-related gene signature predicts glioma cell death and glioma patient progression. *Front. Cell Dev. Biol.* 8:538. 10.3389/fcell.2020.00538 32733879 PMC7363771

[B44] Liu T. Jiang L. Tavana O. Gu W. (2019). The deubiquitylase OTUB1 mediates ferroptosis via stabilization of SLC7A11. *Cancer Res.* 79 1913–1924. 10.1158/0008-5472.Can-18-3037 30709928 PMC6467774

[B45] Ma Y. Yu W. Shrivastava A. Alemi F. Lankachandra K. Srivastava R. K. (2017). Sanguinarine inhibits pancreatic cancer stem cell characteristics by inducing oxidative stress and suppressing sonic hedgehog-Gli-Nanog pathway. *Carcinogenesis* 38 1047–1056. 10.1093/carcin/bgx070 28968696

[B46] Madhavan S. Zenklusen J.-C. Kotliarov Y. Sahni H. Fine H. A. Buetow K. (2009). Rembrandt: helping personalized medicine become a reality through integrative translational research. *Mol. Cancer Res.* 7 157–167. 10.1158/1541-7786.MCR-08-0435 19208739 PMC2645472

[B47] Mantovani A. Marchesi F. Malesci A. Laghi L. Allavena P. (2017). Tumour-associated macrophages as treatment targets in oncology. *Nat. Rev. Clin. Oncol.* 14 399–416. 10.1038/nrclinonc.2016.217 28117416 PMC5480600

[B48] Metselaar D. S. Meel M. H. Benedict B. Waranecki P. Koster J. Kaspers G. J. L. (2019). Celastrol-induced degradation of FANCD2 sensitizes pediatric high-grade gliomas to the DNA-crosslinking agent carboplatin. *EBioMedicine* 50 81–92. 10.1016/j.ebiom.2019.10.062 31735550 PMC6921187

[B49] Mizuno S. Ishida A. (1982). Selective enhancement of the cytotoxicity of the bleomycin derivative, peoplomycin, by local anesthetics alone and combined with hyperthermia. *Cancer Res.* 42 4726–4729. 6181868

[B50] Ngambenjawong C. Gustafson H. H. Pun S. H. (2017). Progress in tumor-associated macrophage (TAM)-targeted therapeutics. *Adv. Drug Deliv. Rev.* 114 206–221. 10.1016/j.addr.2017.04.010 28449873 PMC5581987

[B51] Ostrom Q. T. Bauchet L. Davis F. G. Deltour I. Fisher J. L. Langer C. E. (2014). The epidemiology of glioma in adults: a “state of the science” review. *Neuro. Oncol.* 16 896–913. 10.1093/neuonc/nou087 24842956 PMC4057143

[B52] Ou Y. Wang S. J. Li D. Chu B. Gu W. (2016). Activation of SAT1 engages polyamine metabolism with p53-mediated ferroptotic responses. *Proc. Natl. Acad. Sci. U.S.A.* 113 E6806–E6812. 10.1073/pnas.1607152113 27698118 PMC5098629

[B53] Park A. L. Lin H. K. Yang Q. Sing C. W. Fan M. Mapstone T. B. (2010). Differential expression of type 2 3α/type 5 17β-hydroxysteroid dehydrogenase (AKR1C3) in tumors of the central nervous system. *Int. J. Clin. Exp. Pathol.* 3 743–754. 21151387 PMC2993224

[B54] Picarda E. Ohaegbulam K. C. Zang X. (2016). Molecular pathways: targeting B7-H3 (CD276) for human cancer immunotherapy. *Clin. Cancer Res.* 22 3425–3431. 10.1158/1078-0432.Ccr-15-2428 27208063 PMC4947428

[B55] Romero Y. Wise R. Zolkiewska A. (2020). Proteolytic processing of PD-L1 by ADAM proteases in breast cancer cells. *Cancer Immunol. Immunother.* 69 43–55. 10.1007/s00262-019-02437-2 31796994 PMC6952561

[B56] Santoiemma P. P. Powell D. J. Jr. (2015). Tumor infiltrating lymphocytes in ovarian cancer. *Cancer Biol. Ther.* 16 807–820. 10.1080/15384047.2015.1040960 25894333 PMC4622931

[B57] Schaaf K. Smith S. R. Duverger A. Wagner F. Wolschendorf F. Westfall A. O. (2017). Mycobacterium tuberculosis exploits the PPM1A signaling pathway to block host macrophage apoptosis. *Sci. Rep.* 7:42101. 10.1038/srep42101 28176854 PMC5296758

[B58] Shen L. Zhang R. Sun Y. Wang X. Deng A. M. Bi L. (2014). Overexpression of HSBP1 is associated with resistance to radiotherapy in oral squamous epithelial carcinoma. *Med. Oncol.* 31:990. 10.1007/s12032-014-0990-8 24816843

[B59] Smida T. Bruno T. C. Stabile L. P. (2020). Influence of estrogen on the NSCLC microenvironment: a comprehensive picture and clinical implications. *Front. Oncol.* 10:137. 10.3389/fonc.2020.00137 32133288 PMC7039860

[B60] Stanton S. E. Disis M. L. (2016). Clinical significance of tumor-infiltrating lymphocytes in breast cancer. *J. Immunother. Cancer* 4:59. 10.1186/s40425-016-0165-6 27777769 PMC5067916

[B61] Stockwell B. R. Friedmann Angeli J. P. Bayir H. Bush A. I. Conrad M. Dixon S. J. (2017). Ferroptosis: a regulated cell death nexus linking metabolism, redox biology, and disease. *Cell* 171 273–285. 10.1016/j.cell.2017.09.021 28985560 PMC5685180

[B62] Szklarczyk D. Santos A. von Mering C. Jensen L. J. Bork P. Kuhn M. (2016). STITCH 5: augmenting protein-chemical interaction networks with tissue and affinity data. *Nucleic Acids Res.* 44 D380–D384. 10.1093/nar/gkv1277 26590256 PMC4702904

[B63] Thakur V. S. Aguila B. Brett-Morris A. Creighton C. J. Welford S. M. (2019). Spermidine/spermine N1-acetyltransferase 1 is a gene-specific transcriptional regulator that drives brain tumor aggressiveness. *Oncogene* 38 6794–6800. 10.1038/s41388-019-0917-0 31399646 PMC6786946

[B64] Tian J. Lou J. Cai Y. Rao M. Lu Z. Zhu Y. (2020). Risk SNP-mediated enhancer-promoter interaction drives colorectal cancer through both FADS2 and AP002754.2. *Cancer Res.* 80 1804–1818. 10.1158/0008-5472.Can-19-2389 32127356

[B65] Tibshirani R. Bien J. Friedman J. Hastie T. Simon N. Taylor J. (2012). Strong rules for discarding predictors in lasso-type problems. *J. R. Stat. Soc. Series B Stat. Methodol.* 74 245–266. 10.1111/j.1467-9868.2011.01004.x 25506256 PMC4262615

[B66] von Mering C. Jensen L. J. Snel B. Hooper S. D. Krupp M. Foglierini M. (2005). STRING: known and predicted protein-protein associations, integrated and transferred across organisms. *Nucleic Acids Res.* 33 D433–D437. 10.1093/nar/gki005 15608232 PMC539959

[B67] Wang T. J. C. Mehta M. P. (2019). Low-grade glioma radiotherapy treatment and trials. *Neurosurg. Clin. N. Am.* 30 111–118. 10.1016/j.nec.2018.08.008 30470398

[B68] Wang Z. Wang Z. Zhang C. Liu X. Li G. Liu S. (2018). Genetic and clinical characterization of B7-H3 (CD276) expression and epigenetic regulation in diffuse brain glioma. *Cancer Sci.* 109 2697–2705. 10.1111/cas.13744 30027617 PMC6125452

[B69] Xia X. Fan X. Zhao M. Zhu P. (2019). The relationship between ferroptosis and tumors: a novel landscape for therapeutic approach. *Curr. Gene Ther.* 19 117–124. 10.2174/1566523219666190628152137 31264548 PMC7046989

[B70] Yamaguchi Y. Kasukabe T. Kumakura S. (2018). Piperlongumine rapidly induces the death of human pancreatic cancer cells mainly through the induction of ferroptosis. *Int. J. Oncol.* 52 1011–1022. 10.3892/ijo.2018.4259 29393418

[B71] Yu G. Wang L. G. Han Y. He Q. Y. (2012). Clusterprofiler: an R package for comparing biological themes among gene clusters. *OMICS* 16 284–287. 10.1089/omi.2011.0118 22455463 PMC3339379

[B72] Zang X. Sullivan P. S. Soslow R. A. Waitz R. Reuter V. E. Wilton A. (2010). Tumor associated endothelial expression of B7-H3 predicts survival in ovarian carcinomas. *Mod. Pathol.* 23 1104–1112. 10.1038/modpathol.2010.95 20495537 PMC2976590

[B73] Zhang G. Xu Y. Lu X. Huang H. Zhou Y. Lu B. (2009). Diagnosis value of serum B7-H3 expression in non-small cell lung cancer. *Lung Cancer* 66 245–249. 10.1016/j.lungcan.2009.01.017 19269710

[B74] Zhang R. Wang G. Zhang P. F. Zhang J. Huang Y. X. Lu Y. M. (2017). Sanguinarine inhibits growth and invasion of gastric cancer cells via regulation of the DUSP4/ERK pathway. *J. Cell. Mol. Med.* 21 1117–1127. 10.1111/jcmm.13043 27957827 PMC5431127

[B75] Zhang S. Leng T. Zhang Q. Zhao Q. Nie X. Yang L. (2018). Sanguinarine inhibits epithelial ovarian cancer development via regulating long non-coding RNA CASC2-EIF4A3 axis and/or inhibiting NF-κB signaling or PI3K/AKT/mTOR pathway. *Biomed. Pharmacother.* 102 302–308. 10.1016/j.biopha.2018.03.071 29571014

[B76] Zhao Z. Meng F. Wang W. Wang Z. Zhang C. Jiang T. (2017). Comprehensive RNA-seq transcriptomic profiling in the malignant progression of gliomas. *Sci. Data* 4:170024. 10.1038/sdata.2017.24 28291232 PMC5349247

[B77] Zheng Y. Liao N. Wu Y. Gao J. Li Z. Liu W. (2019). High expression of B7-H2 or B7-H3 is associated with poor prognosis in hepatocellular carcinoma. *Mol. Med. Rep.* 19 4315–4325. 10.3892/mmr.2019.10080 30942404 PMC6472081

