# Supplementary material for: Identification and Validation of an 11-Ferroptosis Related Gene Signature and Its Correlation With Immune Checkpoint Molecules in Glioma
Source: Front Cell Dev Biol. 2021 Jun 23;9:652599. doi: 10.3389/fcell.2021.652599 (PMC8262596; doi:10.3389/fcell.2021.652599)
Supplement: Supplementary file 1 [file Data_Sheet_1.docx]

Table S1. 60 ferroptosis-related genes retrieved from previous studies

| Ferroptosis-related genes | Name |
| --- | --- |
| ACSL4 | acyl-CoA synthetase long-chain family member 4 |
| AKR1C1 | aldo-keto reductase family 1 member C1 |
| AKR1C2 | aldo-keto reductase family 1 member C2 |
| AKR1C3 | aldo-keto reductase family 1 member C3 |
| ALOX15 | [arachidonate 15-lipoxygenase](https://www.ncbi.nlm.nih.gov/gene/246) |
| ALOX5 | [arachidonate 5-lipoxygenase](https://www.ncbi.nlm.nih.gov/gene/240) |
| ALOX12 | [arachidonate 12-lipoxygenase](https://www.ncbi.nlm.nih.gov/gene/240) |
| ATP5MC3 | ATP synthase membrane subunit c locus 3 |
| CARS | cysteinyl tRNA synthetase |
| CBS | cystathionine beta synthase |
| CD44 | CD44 molecule |
| CHAC1 | ChaC glutathione- specific gamma-glutamylcyclotransferase 1 |
| CISD1 | CDGSH iron sulfur domain 1 |
| CS | citrate synthase |
| DPP4 | dipeptidyl-dippeptidase-4 |
| FANCD2 | Fanconi anemia complementation group D2 |
| GCLC | glutamate-cysteine ligase catalytic subunit |
| GCLM | glutamate-cysteine ligase modifier subunit |
| GLS2 | glutaminase 2 |
| GPX4 | glutathione peroxidase 4 |
| GSS | glutathione synthetase |
| HMGCR | 3-hydroxy-3- methylglutaryl-CoA reductase |
| HSPB1 | heat shock protein beta 1 |
| CRYAB | heat shock protein beta 5 |
| LPCAT3 | lysophosphatidylcholine acyltransferase 3 |
| MT1G | metallothionein-1G |
| NCOA4 | nuclear receptor coactivator 4 |
| PTGS2 | prostaglandin-endoperoxide synthase 2 |
| RPL8 | ribosomal protein L8 |
| SAT1 | spermidine/spermine N1-acetyltransferase 1 |
| SLC7A11 | solute carrier family 7 member 11 |
| FDFT1 | farnesyl-diphosphate farnesyltransferase 1 |
| TFRC | transferrin receptor |
| TP53 | tumor protein 53 |
| EMC2 | ER membrane protein complex subunit 2 |
| AIFM2 | apoptosis inducing factor mitochondria associated 2 |
| PHKG2 | phosphorylase kinase, g2 |
| HSBP1 | heat-shock 27-k Da protein 1 |
| ACO1 | aconitase 1 |
| FTH1 | ferritin heavy chain 1 |
| STEAP3 | six-transmembrane epithelial antigen of prostate 3 |
| NFS1 | NFS1 Cysteine Desulfurase |
| ACSL3 | acyl-CoA synthetase long-chain family member 3 |
| ACACA | Acetyl-CoA carboxylase alpha |
| PEBP1 | phosphatidylethanolamine-binding protein 1 |
| ZEB1 | zinc finger E-box-binding homeobox 1 |
| SQLE | squalene monooxygenase |
| FADS2 | fatty acid desaturase 2/acyl-CoA 6-desaturase |
| NFE2L2 | nuclear factor, erythroid 2 like 2 |
| KEAP1 | kelch-like ECH- associated protein 1 |
| NQO1 | quinone oxidoreductase-1 |
| NOX1 | NADPH oxidase 1 |
| ABCC1 | ATP binding cassette subfamily C member 1 |
| SLC1A5 | solute carrier family 1 member 5 |
| GOT1 | glutamic-oxaloacetic transaminase 1 |
| G6PD | glucose-6-phosphate dehydrogenase |
| PGD | phosphoglycerate dehydrogenase |
| IREB2 | iron response element-binding protein 2 |
| HMOX1 | heme oxygenase 1 |
| ACSF2 | acyl-CoA synthetase family member 2 |

Table S2. Enrichment analysis of downstream target genes of the candidate drugs

| pathway ID | Pathway description | Observed gene count | False discovery rate | Matching proteins (IDs) |
| --- | --- | --- | --- | --- |
| GO.0003707 | steroid hormone receptor activity | 6 | 4.69E-06 | AR,ESR1,ESR2,ESRRA,NR3C1,PGR |
| GO.0005496 | steroid binding | 6 | 2.26E-05 | AR,ESR1,ESR2,ESRRA,NR3C1,PGR |
| GO.0004879 | RNA polymerase II transcription factor activity, ligand-activated sequence-specific DNA binding | 5 | 3.65E-05 | AR,ESR1,ESR2,ESRRA,PGR |
| GO.0034056 | estrogen response element binding | 2 | 0.0184 | ESR1,ESR2 |
| GO.0030284 | estrogen receptor activity | 2 | 0.0255 | ESR1,ESR2 |
| GO.0000976 | transcription regulatory region sequence-specific DNA binding | 7 | 0.0279 | AR,ESR1,ESR2,ESRRA,HDAC1,NR3C1,PGR |
| GO.0001077 | transcriptional activator activity, RNA polymerase II core promoter proximal region sequence-specific binding | 5 | 0.0335 | AR,ESR1,ESRRA,NR3C1,PGR |
| GO.0043401 | steroid hormone mediated signaling pathway | 6 | 1.96E-05 | AR,ESR1,ESR2,ESRRA,NR3C1,PGR |
| GO.0030518 | intracellular steroid hormone receptor signaling pathway | 5 | 0.000839 | AR,ESR1,ESR2,NR3C1,PGR |
| GO.0071383 | cellular response to steroid hormone stimulus | 6 | 0.000839 | AR,ESR1,ESR2,ESRRA,NR3C1,PGR |
| GO.0006367 | transcription initiation from RNA polymerase II promoter | 7 | 0.00116 | AR,ESR1,ESR2,ESRRA,HDAC1,NR3C1,PGR |
| GO.0030522 | intracellular receptor signaling pathway | 6 | 0.00244 | AR,ESR1,ESR2,ESRRA,NR3C1,PGR |
| GO.0071407 | cellular response to organic cyclic compound | 7 | 0.00635 | AR,ESR1,ESR2,ESRRA,KCNJ11,NR3C1,PGR |
| GO.0048545 | response to steroid hormone | 7 | 0.0128 | AR,ESR1,ESR2,ESRRA,KCNJ11,NR3C1,PGR |
| IPR000536 | Nuclear hormone receptor, ligand-binding domain | 5 | 2.25E-05 | AR,ESR1,ESR2,ESRRA,NR3C1 |
| IPR001628 | Zinc finger, nuclear hormone receptor-type | 5 | 2.25E-05 | AR,ESR1,ESR2,ESRRA,NR3C1 |
| IPR013088 | Zinc finger, NHR/GATA-type | 5 | 2.25E-05 | AR,ESR1,ESR2,ESRRA,NR3C1 |
| IPR024178 | Oestrogen receptor/oestrogen-related receptor | 3 | 4.42E-05 | ESR1,ESR2,ESRRA |
| IPR001723 | Nuclear hormone receptor | 4 | 0.000712 | ESR1,ESR2,ESRRA,NR3C1 |
| PF00104 | Ligand-binding domain of nuclear hormone receptor | 5 | 1.19E-05 | AR,ESR1,ESR2,ESRRA,NR3C1 |
| PF00105 | Zinc finger, C4 type (two domains) | 5 | 1.19E-05 | AR,ESR1,ESR2,ESRRA,NR3C1 |

**
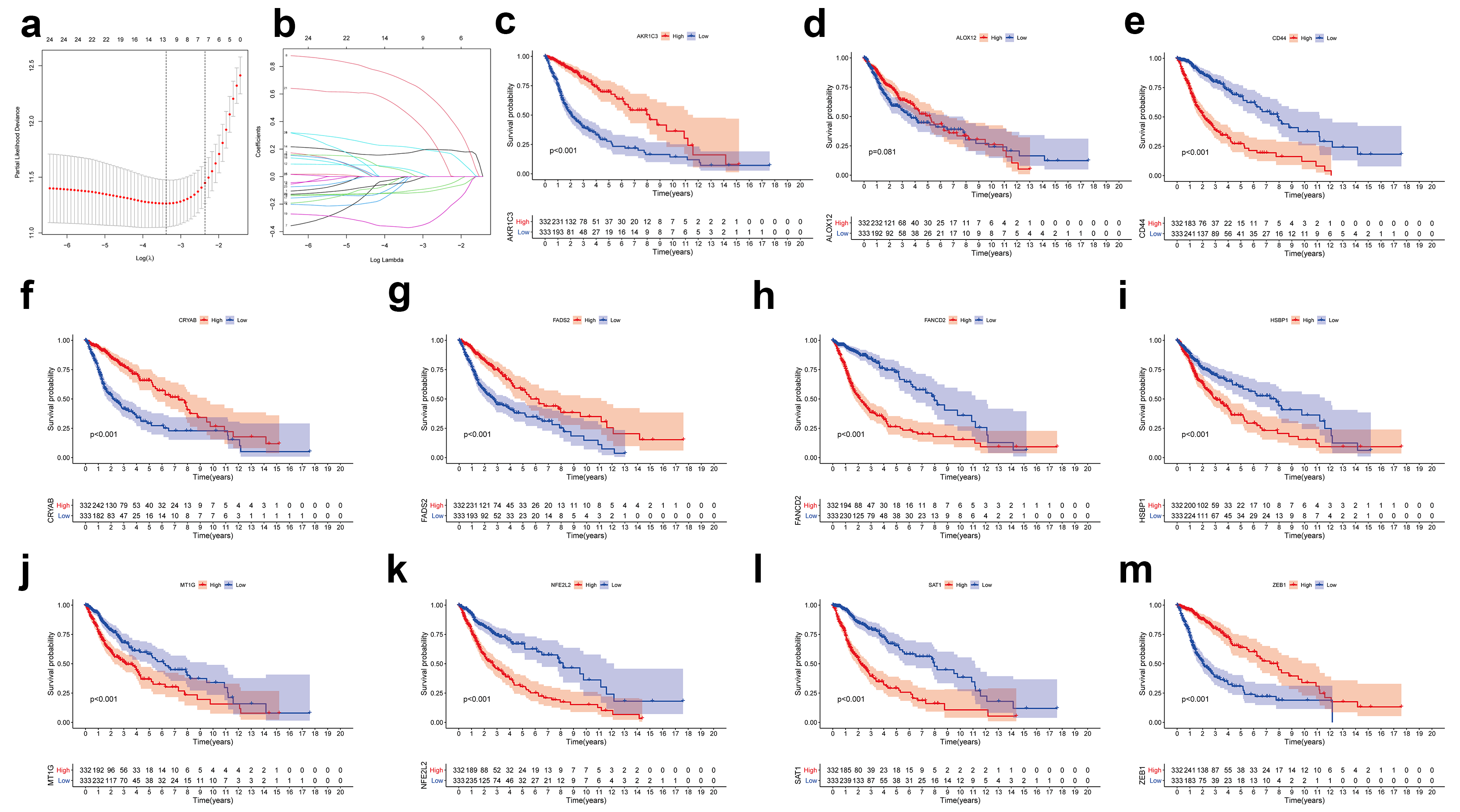
**

**Figure S1. Prognostic analysis of the 11-gene signature model in the TCGA dataset. (a) and (b)** A 11-gene signature was identified based on LASSO Cox regression. **(c-m)** Survival analyses of the 11 ferroptosis genes.


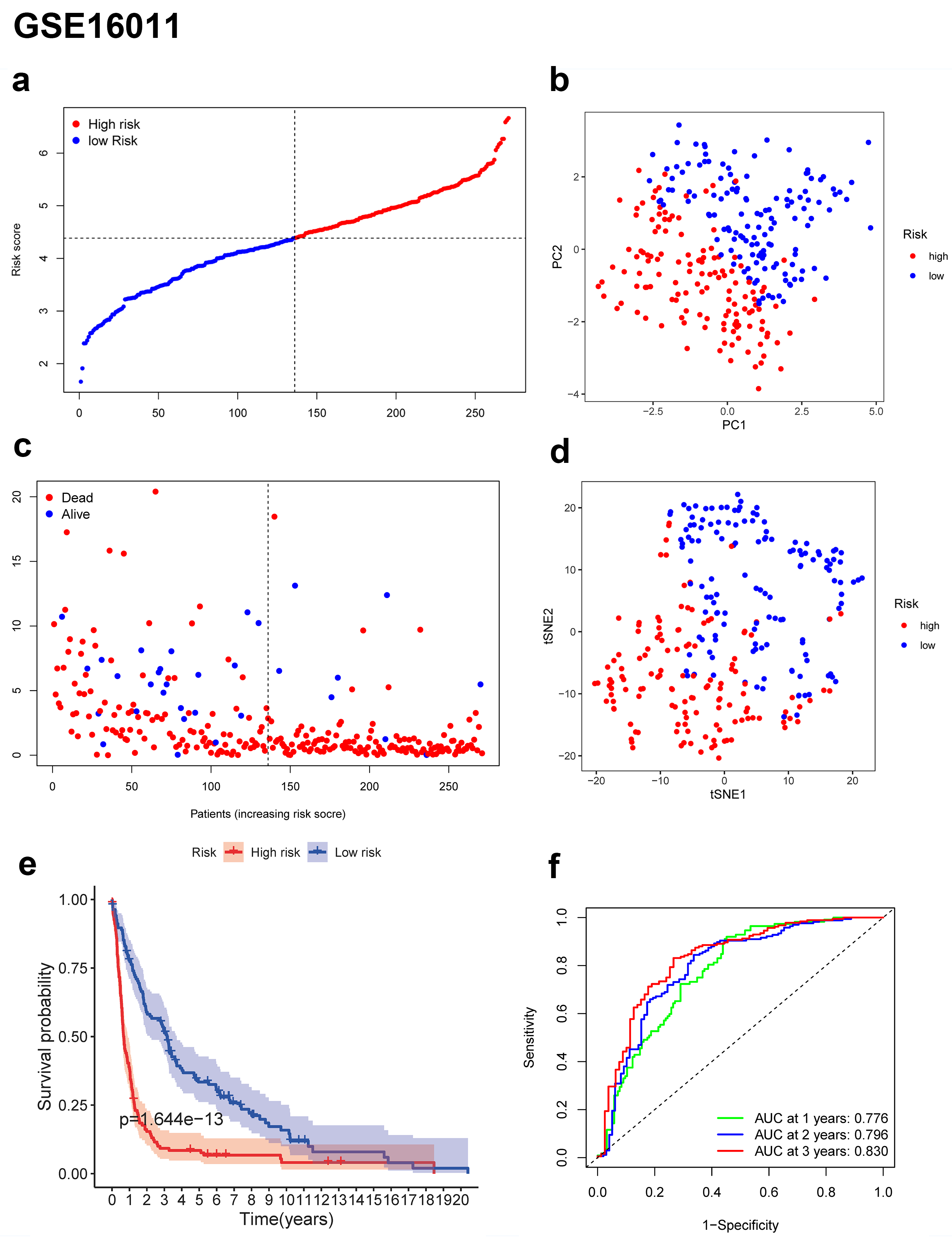


**Figure S2. Validation of the 11-gene signature in the GSE16011 cohort. (a,c)** Distribution and median value of risk scores in the GSE16011 cohort. **(b)** PCA plot of the GSE16011 cohort. **(d)** t-SNE analysis of the GSE16011 cohort. **(e)** Kaplan-Meier survival curve for the OS of patients in the high-risk group (red line) and low-risk group (blue line) in the GSE16011 cohort. **(f)** AUC of time-dependent ROC curve analysis in the GSE16011 cohort.


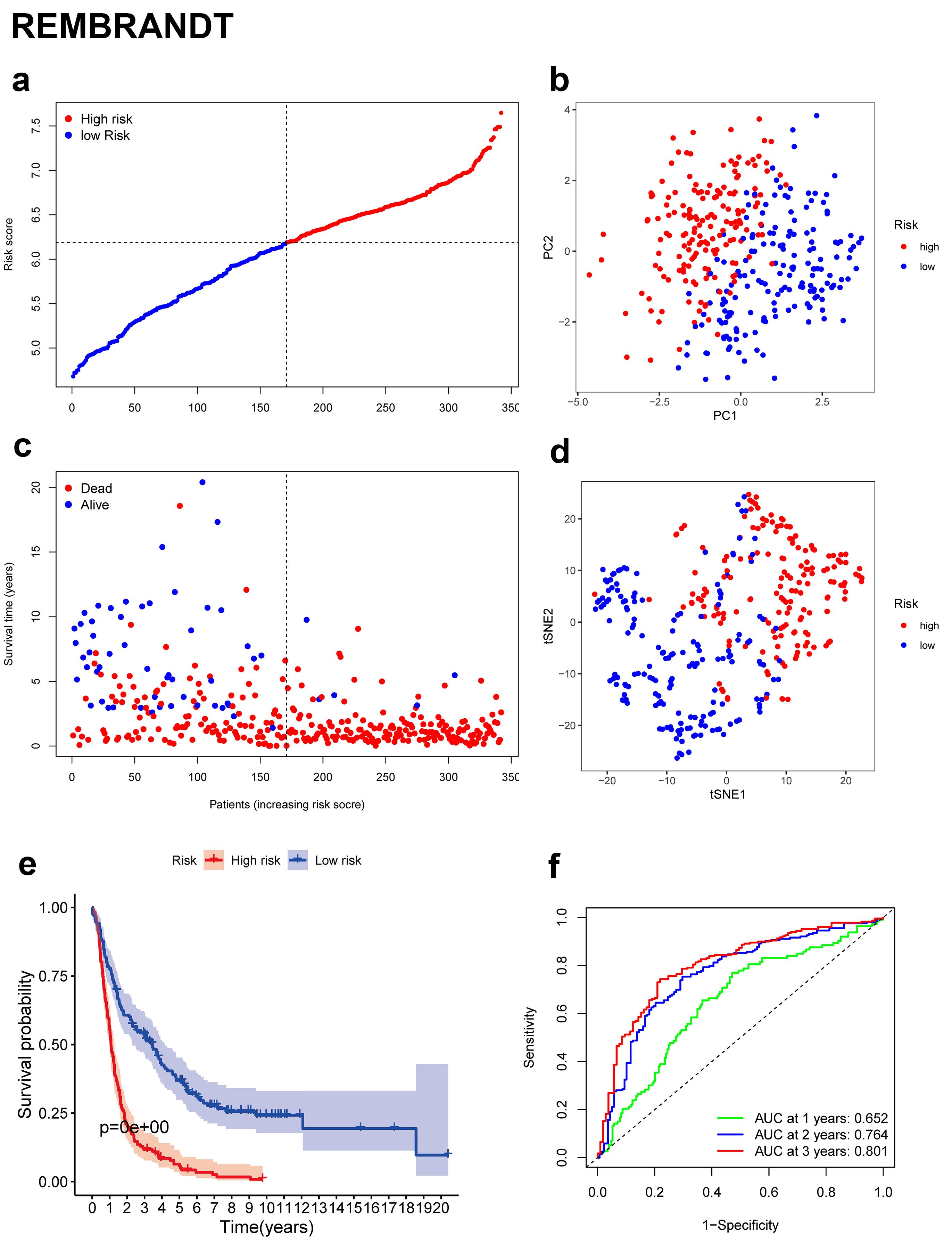


**Figure S3. Validation of the 11-gene signature in the REMBRANDT cohort. (a,c)** Distribution and median value of risk scores in the REMBRANDT cohort. **(b)** PCA plot of the REMBRANDT cohort. **(d)** t-SNE analysis of the REMBRANDT cohort. **(e)** Kaplan-Meier survival curve for the OS of patients in the high-risk group (red line) and low-risk group (blue line) in the REMBRANDT cohort. **(f)** AUC of time-dependent ROC curve analysis in the REMBRANDT cohort.


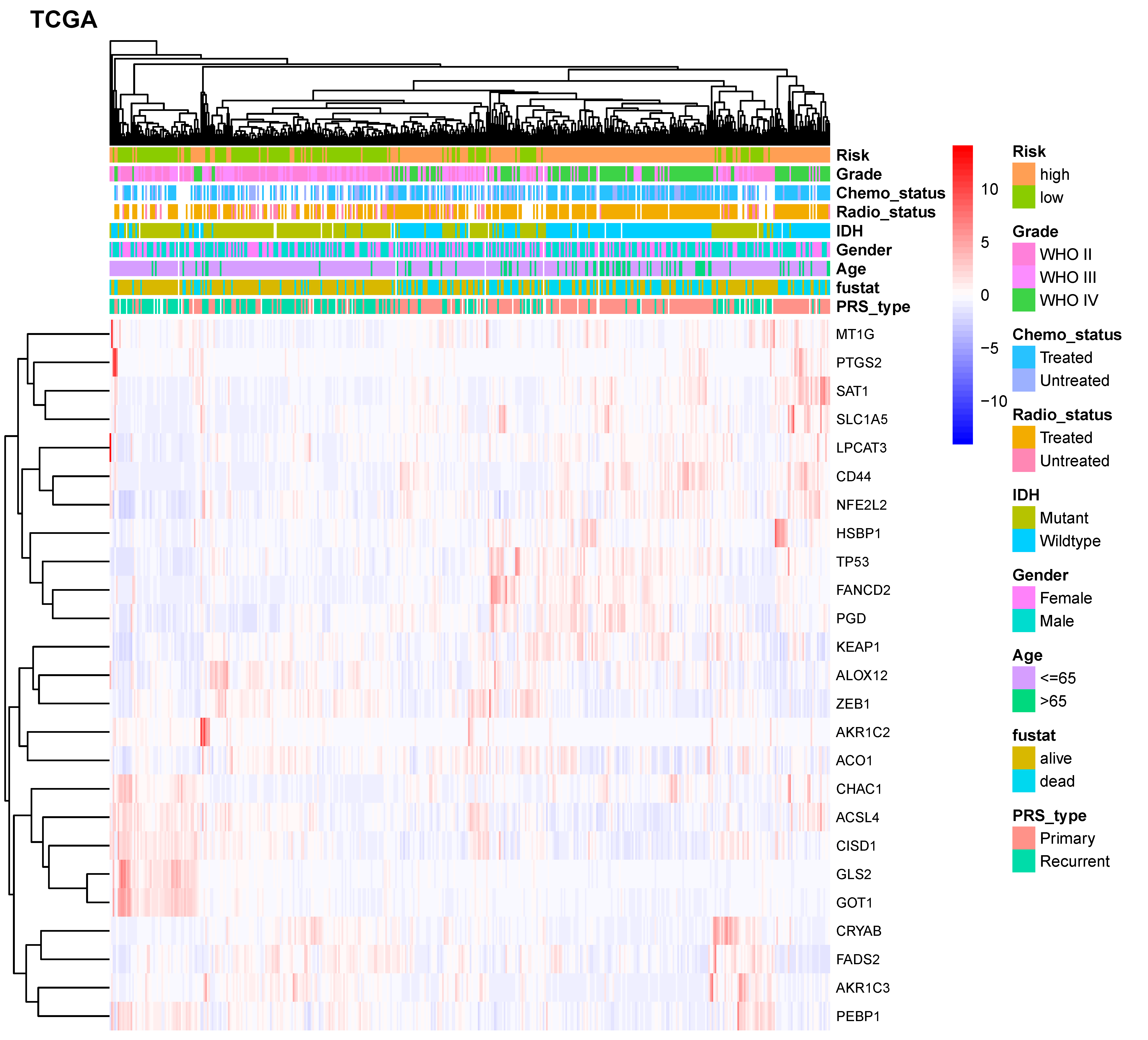


**Figure S4.** Hierarchical clustering showing the correlation between signature risk score, different expression levels of selected ferroptosis-related genes, and clinical or molecular features in the TCGA dataset.


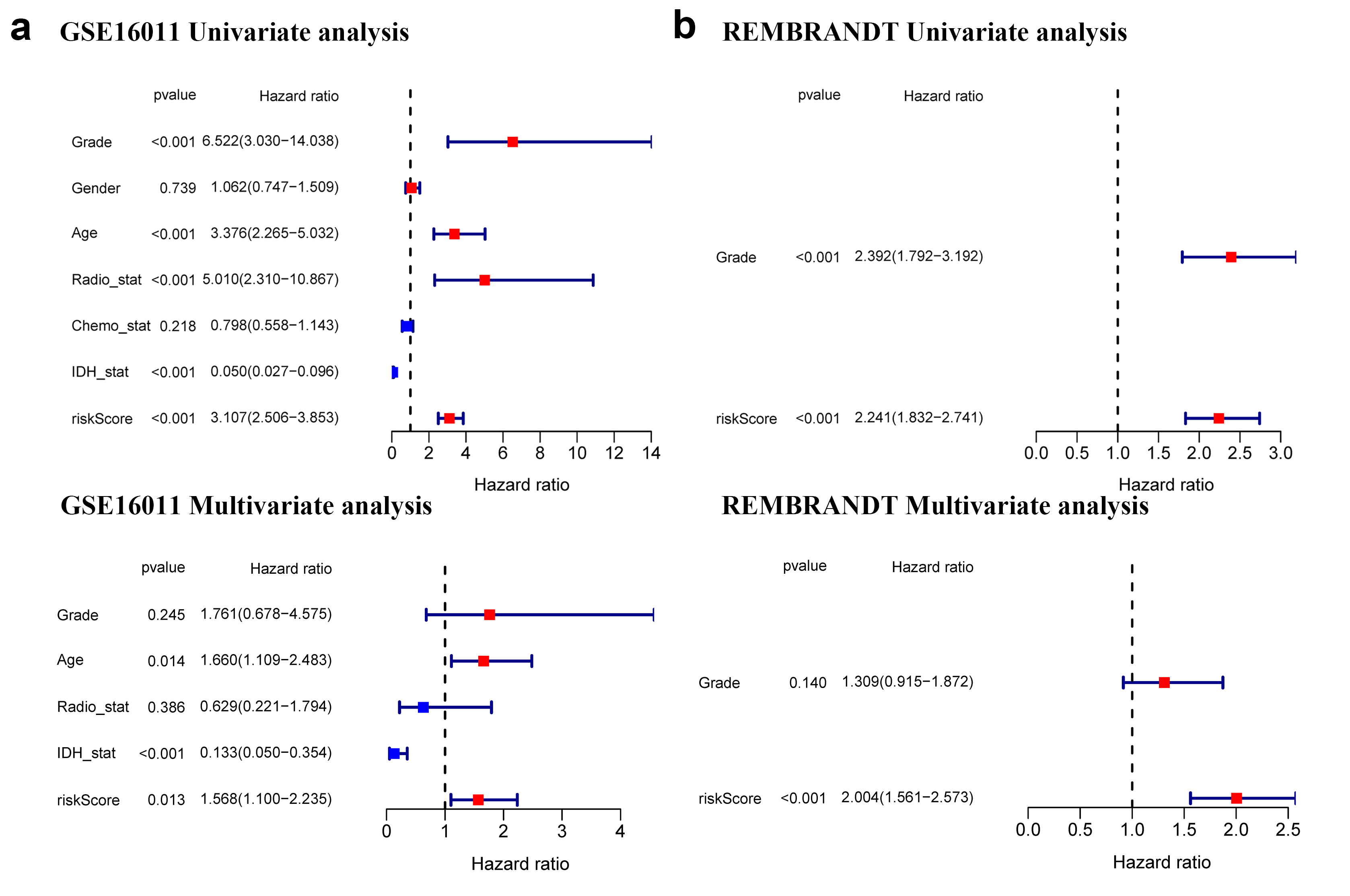


**Figure S5. Independent prognostic value of the 11-gene signature in the GSE16011 and REMBRANDT cohorts. (a)** Univariate and multivariate Cox regression analyses of the signature in the GSE16011 cohort. **(b)** Univariate and multivariate Cox regression analyses of the signature in the REMBRANDT cohort.

**
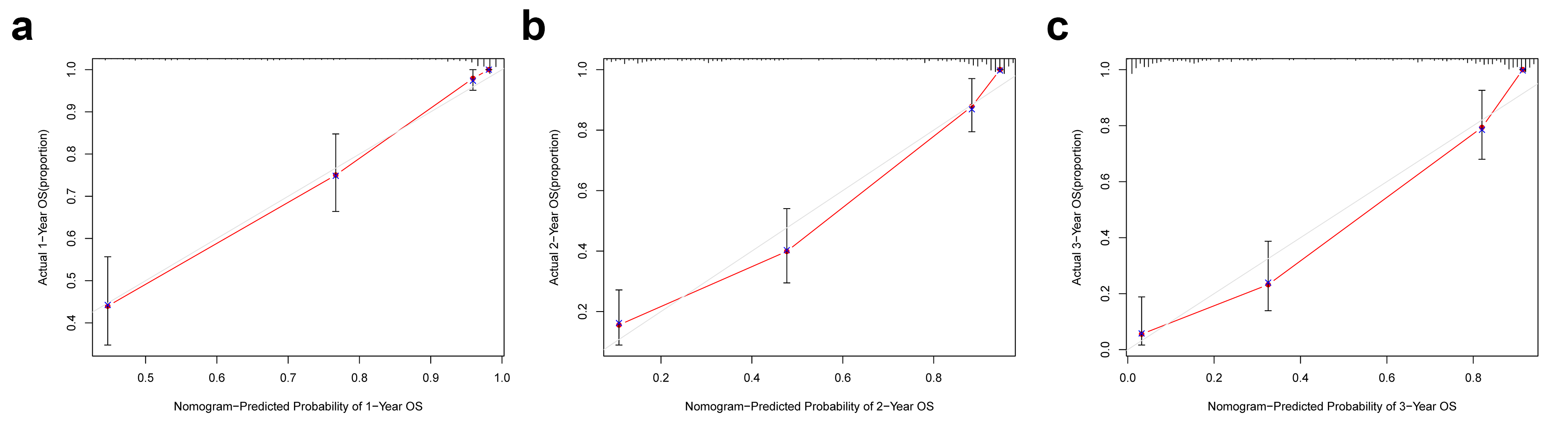
**

**Figure S6. The calibration curve for the nomogram-predicted probability of 1(a), 2(b) and 3(c)-year OS in the TCGA dataset.**

**
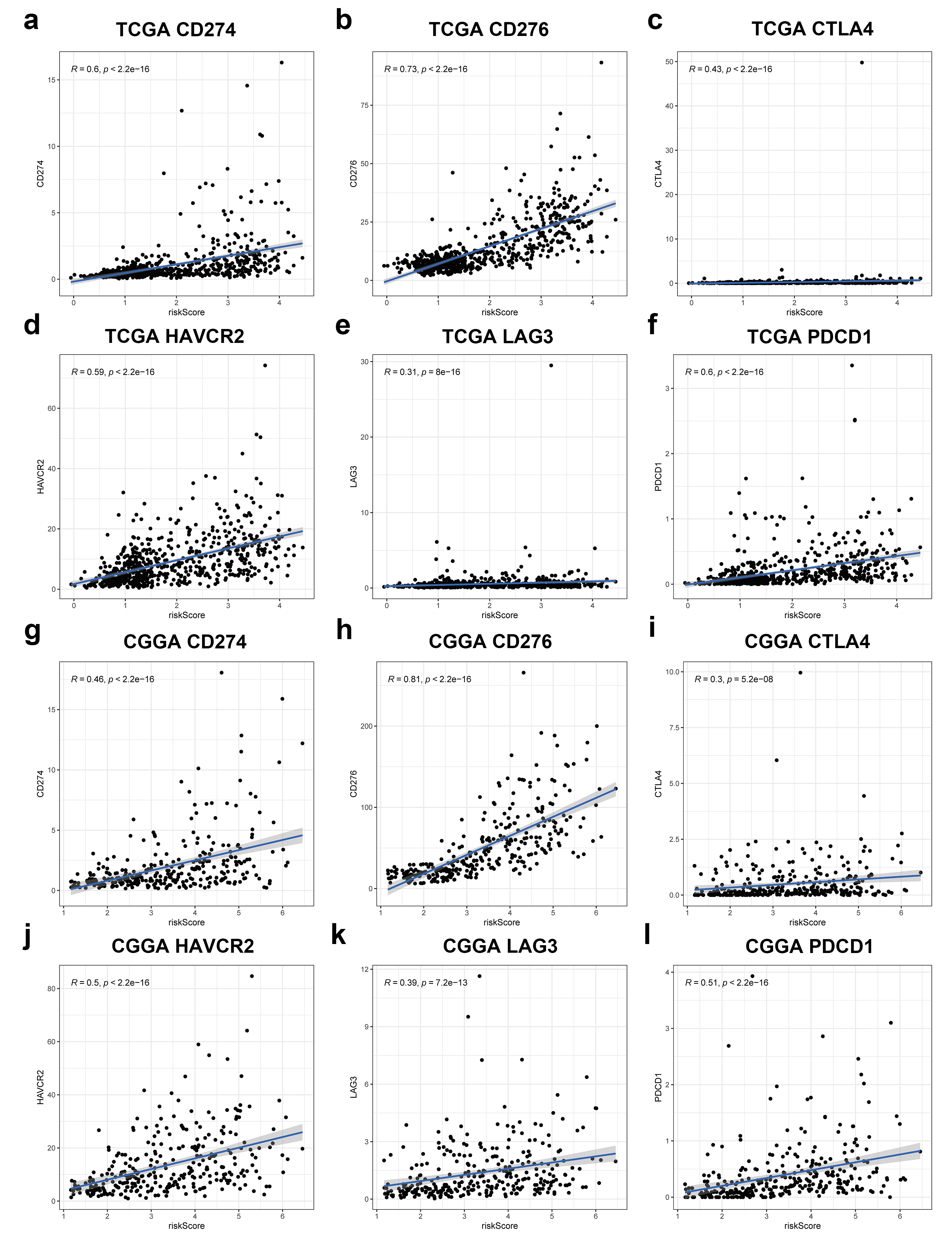
**

**Figure S7. The results of correlation analysis between immune checkpoint related genes and risk scores in the TCGA cohort and the CGGA cohort.** The correlation between the expression of immune checkpoint molecules and risk score in each patient was analyzed using Spearman's correlation coefficient. Results were displayed for both TCGA cohort (a-f) and CGGA cohort (g-l).


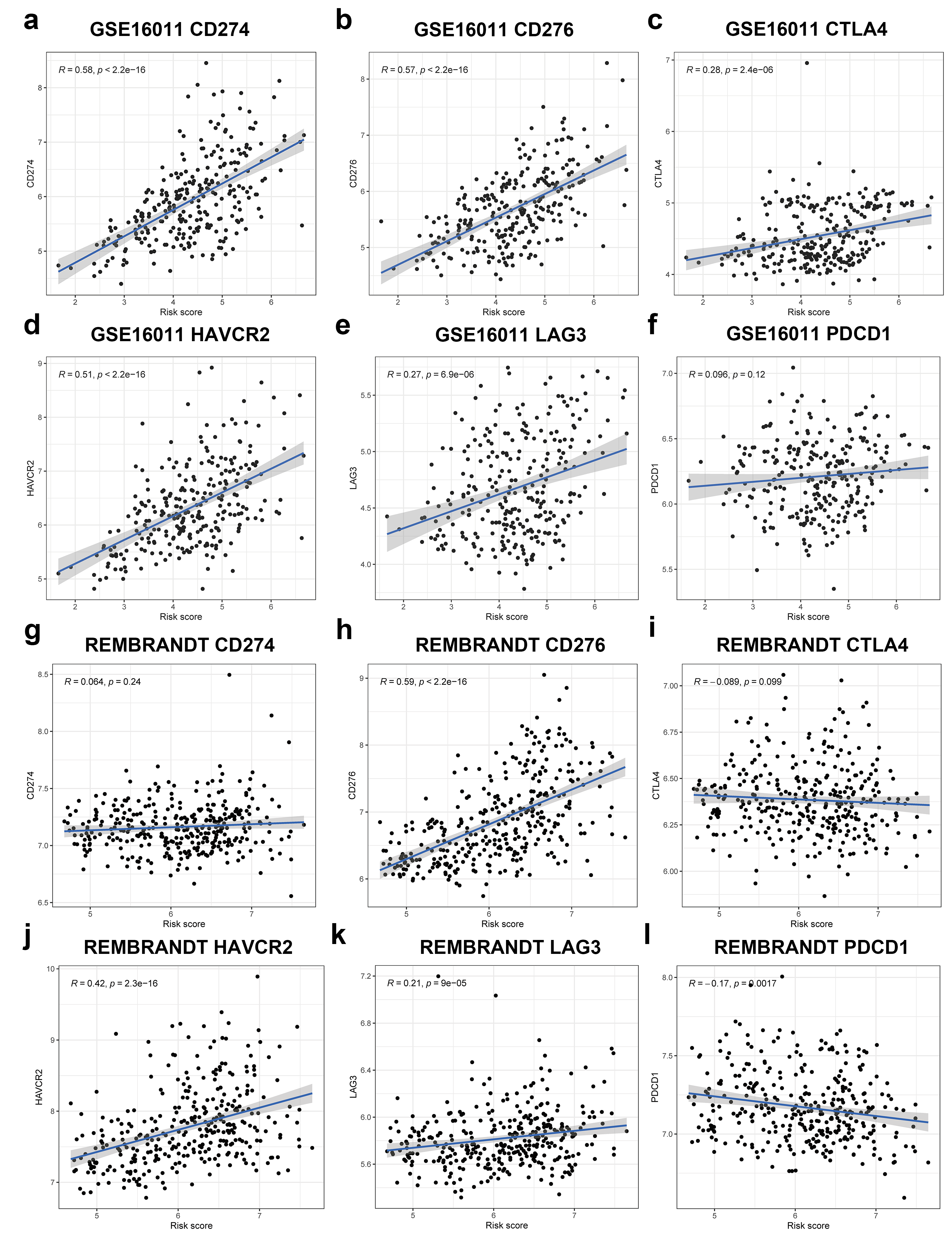


**Figure S8. The results of correlation analysis between immune checkpoint related genes and risk scores in the GSE16011 cohort and the REMBRANDT cohort.** The correlation between the expression of immune checkpoint molecules and risk score in each patient was analyzed using Spearman's correlation coefficient. Results were displayed for both GSE16011 cohort (a-f) and REMBRANDT cohort (g-l).


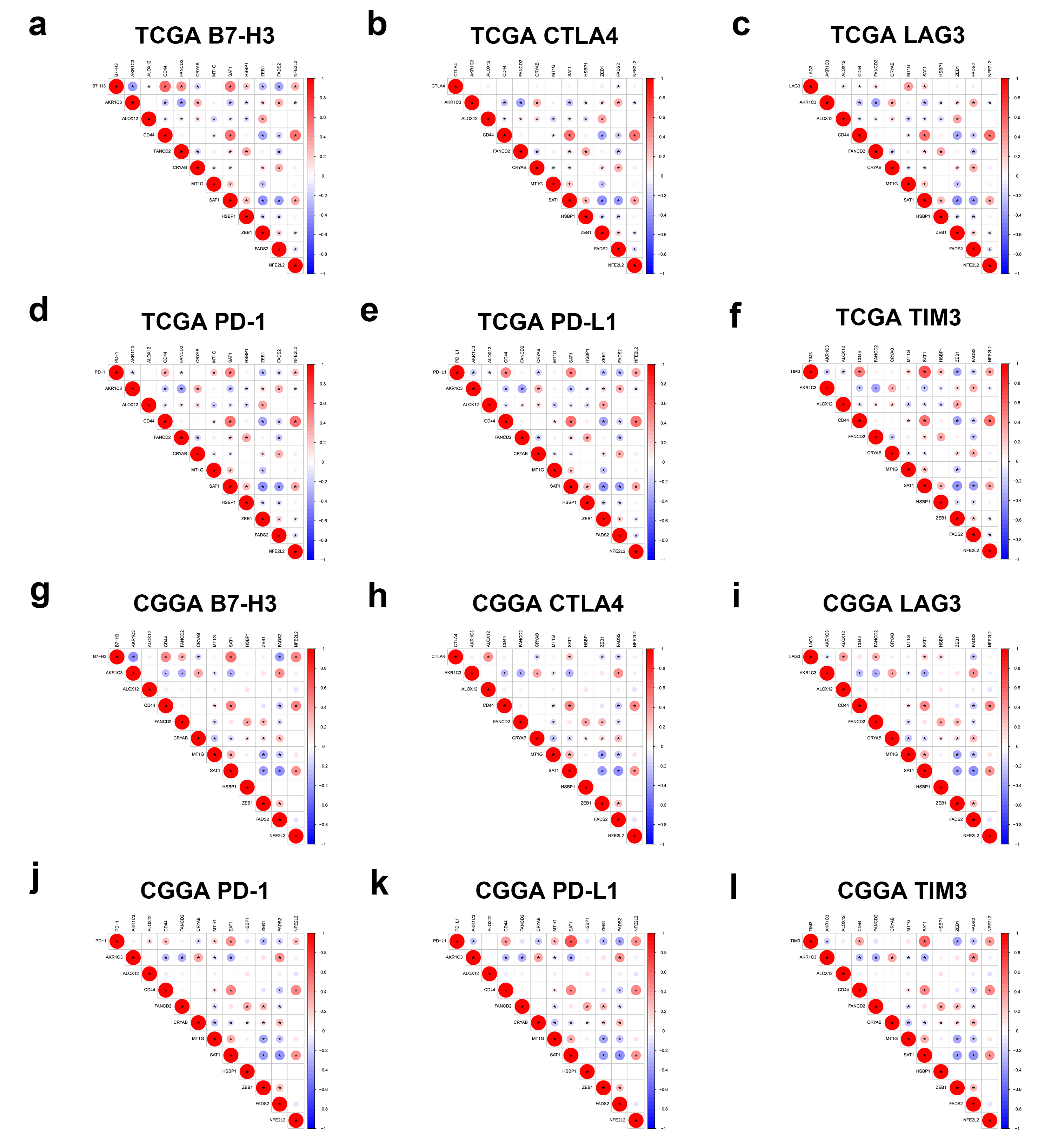


**Figure S9. Correlation analysis of ferroptosis-related genes and immune checkpoint genes in the TCGA(a-f) and the CGGA(g-l) datasets.** Key genes in the TCGA dataset are CD44, FANCD2 and SAT1. Key genes in the CGGA dataset are AKR1C3, ALOX12, CD44, SAT1 and NFE2L2. The common critical genes are CD44 and SAT1. |r|＞0.4.


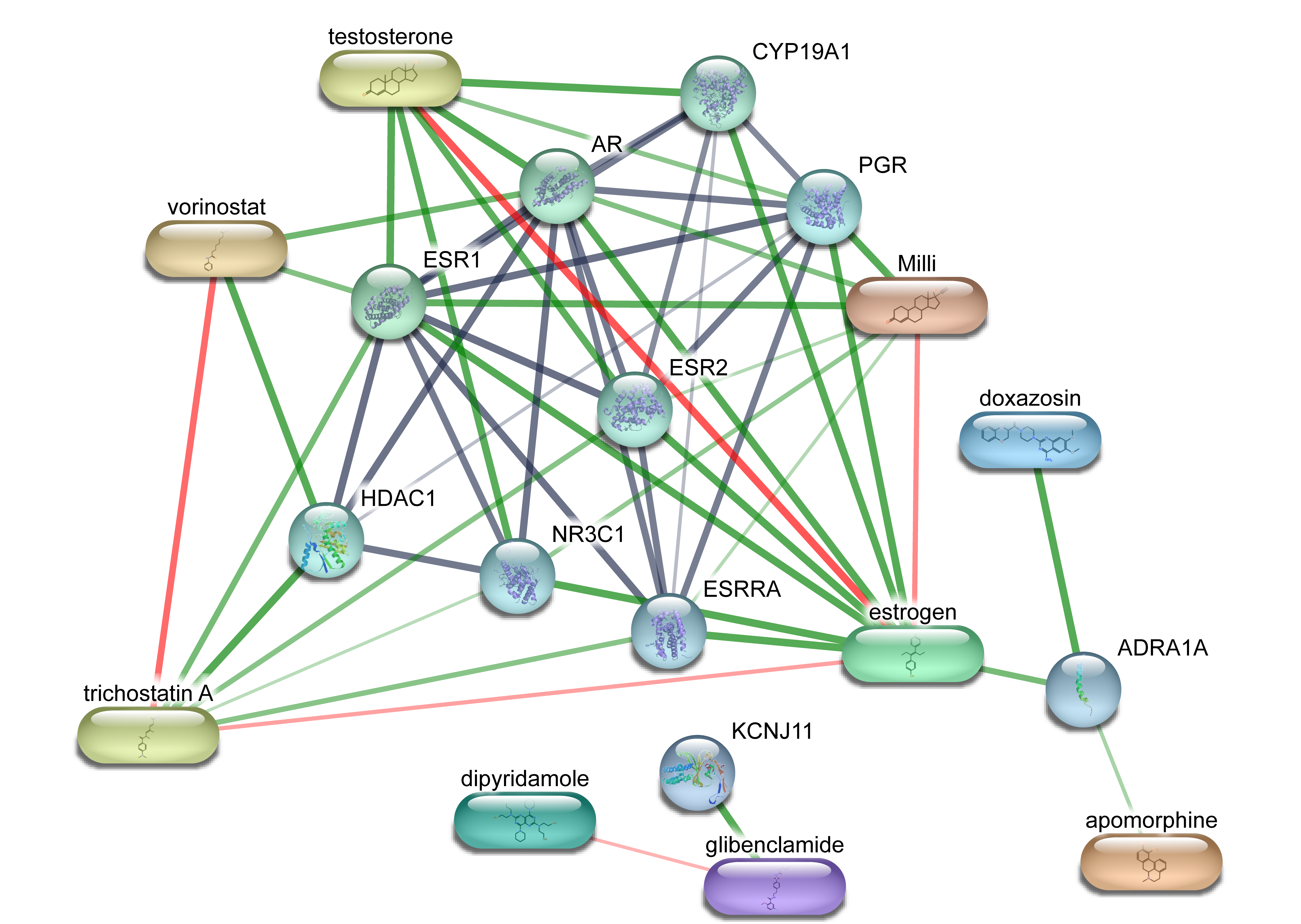


**Figure S10. The network of protein and drug interactions by STITCH database.** The weight of edges represents the confidence score. A thicker line represents a stronger correlation. Protein-protein interactions are depicted in grey. Chemical-chemical interactions are depicted in red. Chemical-protein interactions are depicted in green.

**
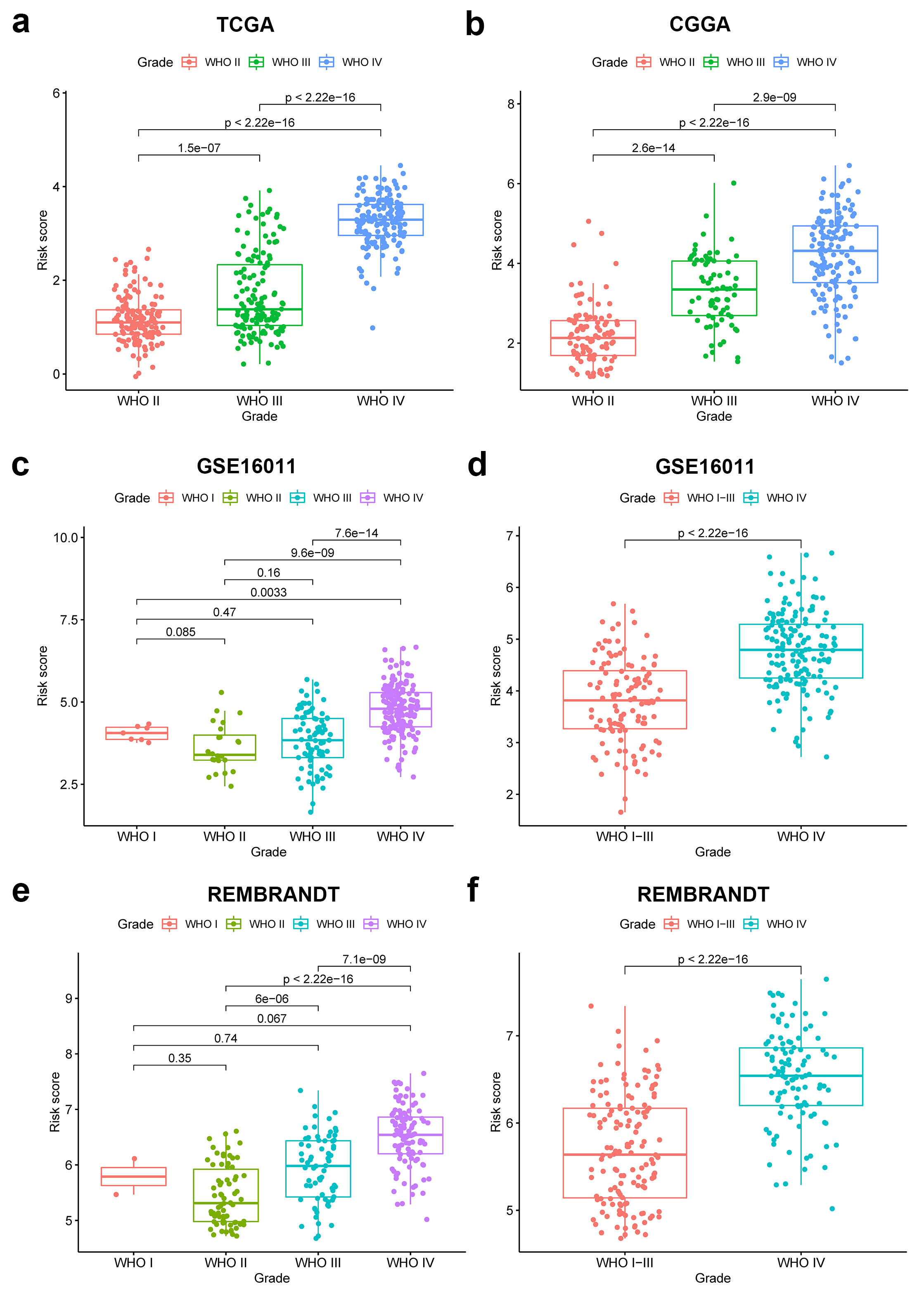
**

**Figure S11. Correlation between risk score and glioma grade in the TCGA(a), CGGA(b), GSE16011(c-d), and REMBRANDT(e-f) datasets.**

**
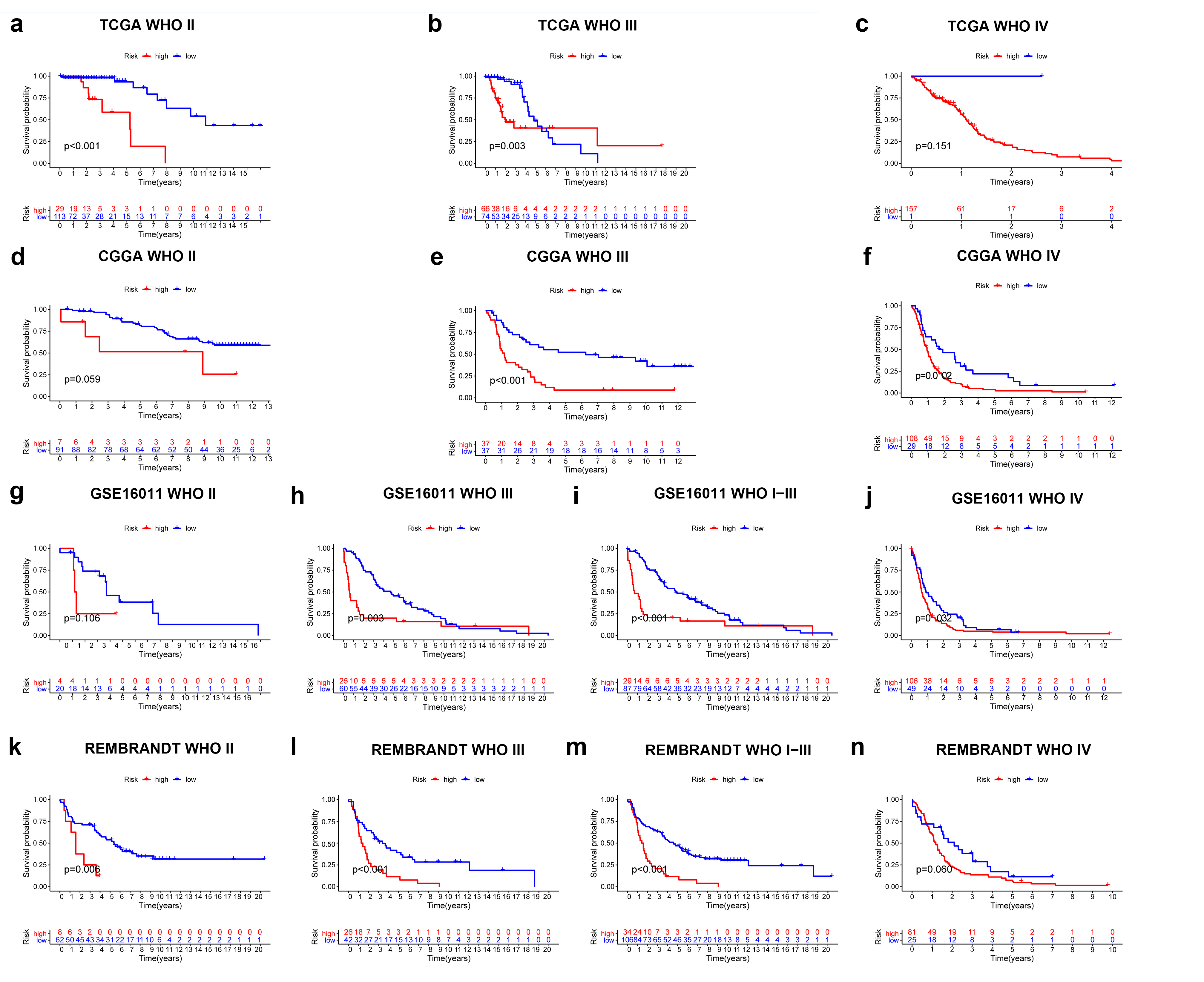
**

**Figure S12. Kaplan-Meier curves for low-risk and high-risk patients classified as WHO grade II to IV in the TCGA(a-c), CGGA(d-f), GSE16011(g-j), and REMBRANDT(k-n) datasets.**
